# Global Sequestration Potential of Increased Organic Carbon in Cropland Soils

**DOI:** 10.1038/s41598-017-15794-8

**Published:** 2017-11-14

**Authors:** Robert J. Zomer, Deborah A. Bossio, Rolf Sommer, Louis V. Verchot

**Affiliations:** 1 Key Laboratory for Plant Diversity and Biogeography of East Asia (KLPB), Kunming Institute of Botany, Chinese Academy of Science, Kunming, 650201 Yunnan China; 2 The Nature Conservatory (TNC), Santa Cruz, California USA; 3 International Center for Tropical Agriculture (CIAT), Nairobi, Kenya; 4 International Center for Tropical Agriculture (CIAT), Cali, Colombia

**Keywords:** Ecological modelling, Climate-change mitigation, Agroecology, Ecosystem services

## Abstract

The role of soil organic carbon in global carbon cycles is receiving increasing attention both as a potentially large and uncertain source of CO_2_ emissions in response to predicted global temperature rises, and as a natural sink for carbon able to reduce atmospheric CO_2_. There is general agreement that the technical potential for sequestration of carbon in soil is significant, and some consensus on the magnitude of that potential. Croplands worldwide could sequester between 0.90 and 1.85 Pg C/yr, i.e. 26–53% of the target of the “4p1000 Initiative: Soils for Food Security and Climate”. The importance of intensively cultivated regions such as North America, Europe, India and intensively cultivated areas in Africa, such as Ethiopia, is highlighted. Soil carbon sequestration and the conservation of existing soil carbon stocks, given its multiple benefits including improved food production, is an important mitigation pathway to achieve the less than 2 °C global target of the Paris Climate Agreement.

## Introduction

Historical and ongoing increase of agricultural production worldwide has profoundly impacted global carbon, water and nutrient cycles^1–4^. Both land-use change to agriculture and agricultural production have and continue to contribute significantly to the increase in atmospheric carbon dioxide (CO_2_), accounting for as much as 24% of global greenhouse gas (GHG) emissions^5^. Almost 50% of all potentially vegetated land surface globally has been converted to croplands, pastures and rangelands^1–4^. This land-use change and soil cultivation have contributed 136 ± 55 petagrams of carbon (Pg C) to the atmosphere from change in biomass carbon since the beginning of the Industrial Revolution, with depletion of soil organic carbon (SOC) accounting for a further contribution of 78 ± 12 Pg C. This estimated 214 ± 67 Pg C from the land-use sector compares to the estimated 270 ± 30 Pg of C contributed by fossil fuel combustion^6^ as a historical carbon source. More recently soil organic matter also has gotten increasing attention as a potentially large and uncertain source of carbon to the atmosphere in the future in response to predicted global temperature rises^7,8^.

Soils, however, can act as both sources and sinks of carbon, depending upon management, biomass input levels, micro-climatic conditions, and bioclimatic change. Substantially more carbon is stored in the world’s soils than is present in the atmosphere. The global soil carbon (C) pool to one-meter depth, estimated at 2500 Pg C, of which about 1500 Pg C is soil organic carbon (SOC), is about 3.2 times the size of the atmospheric pool and 4 times that of the biotic pool^6,9,10^. An extensive body of research has shown that land management practices can increase soil carbon stocks on agricultural lands with practices including addition of organic manures, cover cropping, mulching, conservation tillage, fertility management, agroforestry, and rotational grazing^11,12^. There is general agreement that the technical potential for sequestration of carbon in soil is significant, and some consensus on the magnitude of that potential^13^. On this basis, the 4p1000 initiative on Soil for Food Security and Climate^14^, officially launched by the French Ministry of Agriculture at the United Nations Framework Convention for Climate Change: Conference of the Parties (UNFCCC COP 21) in Paris, aims to sequester approximately 3.5Gt C annually in soils. Croplands will be extremely important in this effort, as these lands are already being actively managed, and so amenable to implementation of improved practices^12^. Furthermore, because almost all cropped soils have lost a large percentage of their pre-cultivation SOC^6,15^, they potentially represent a large sink to re-absorb carbon through the introduction and adoption of improved or proper management aimed towards increased SOC. However, carbon is rarely stored in soils in its elemental form, but rather in the form of organic matter which contains significant amounts of other nutrients, above all nitrogen. Nutrients, biomass productivity, the type of vegetation and water availability, among other constraints therefore can be major limiting factors inhibiting increases in soil carbon sequestration^16^. Further imperative to sequester carbon in soils arises from the multiple co-benefits that are obtained from sequestration of carbon in soils that have been depleted of their organic matter^17^. Soil fertility, health, and functioning are immediate consequences of the amount of soil organic matter (and hence carbon) a soil contains; this is even more important for highly weathered soils, as is the case for the majority of soils in the humid lowland tropics. Increasing carbon in soils also means improving its physical properties and related ecosystems services, such as better water infiltration, water holding capacity, as well as potentially increasing agricultural productivity and ecological resilience^11,12^.

In this analysis, we illustrate where carbon might be sequestered, and how much, if, through improved practices and management, we could increase SOC on agricultural land by a generally accepted (as attainable) moderate to optimistic amount, based on the medium and high sequestration scenarios of Sommer and Bossio (2014). These scenarios from Sommer and Bossio (2014) resulted in an 0.27 and 0.54% increase in SOC in the top 30 cm of soils after 20 years, for the medium and high scenarios, respectively, that is, a 0.012 to 0.027% annual increase. The low scenario in Sommer and Bossio (2014) was not used because it refers to sequestration rates estimated primarily for unimproved pasture land. An implicit basic assumption is that in general, 50 to 70% of soil carbon stocks have been lost in cultivated soils^6,15,17^, such that the SOC status of almost all cultivated soils can be increased. It is expected that these cropped soils will be able to sequester carbon for at least 20 years before reaching saturation points and new SOC equilibriums^13,18^, while meta-analysis of field studies^14^ suggests that in some instances significant sequestration can continue for 30 or even up to 40 years before reaching new equilibriums. We used the recently released ISRIC SoilGrids250m^19^ global database of soil information, to identify and derive basic soil characteristics, i.e. SOC and soil bulk density, and the FAO GLC-Share Land Cover database^20^ to identify and calculate areal extent of the cropland landcover class. The analysis gives a spatially articulated estimate of the distribution and increase of SOC if equal sequestration is reached, within the medium and high scenarios, on all available cropland soils through improved practices. The results of this paper provide an estimate of what the potential amount of sequestered carbon would be in terms of tons of carbon per hectare, spatially articulated at 250 m resolution, and in terms of Pg C regionally and globally, allowing for a quantified discussion of the importance of this carbon pool within on-going global discussions regarding mitigation potential within the agricultural sector.

## Results

### Global Soil Organic Carbon Stocks on Croplands

**Figure 1:**
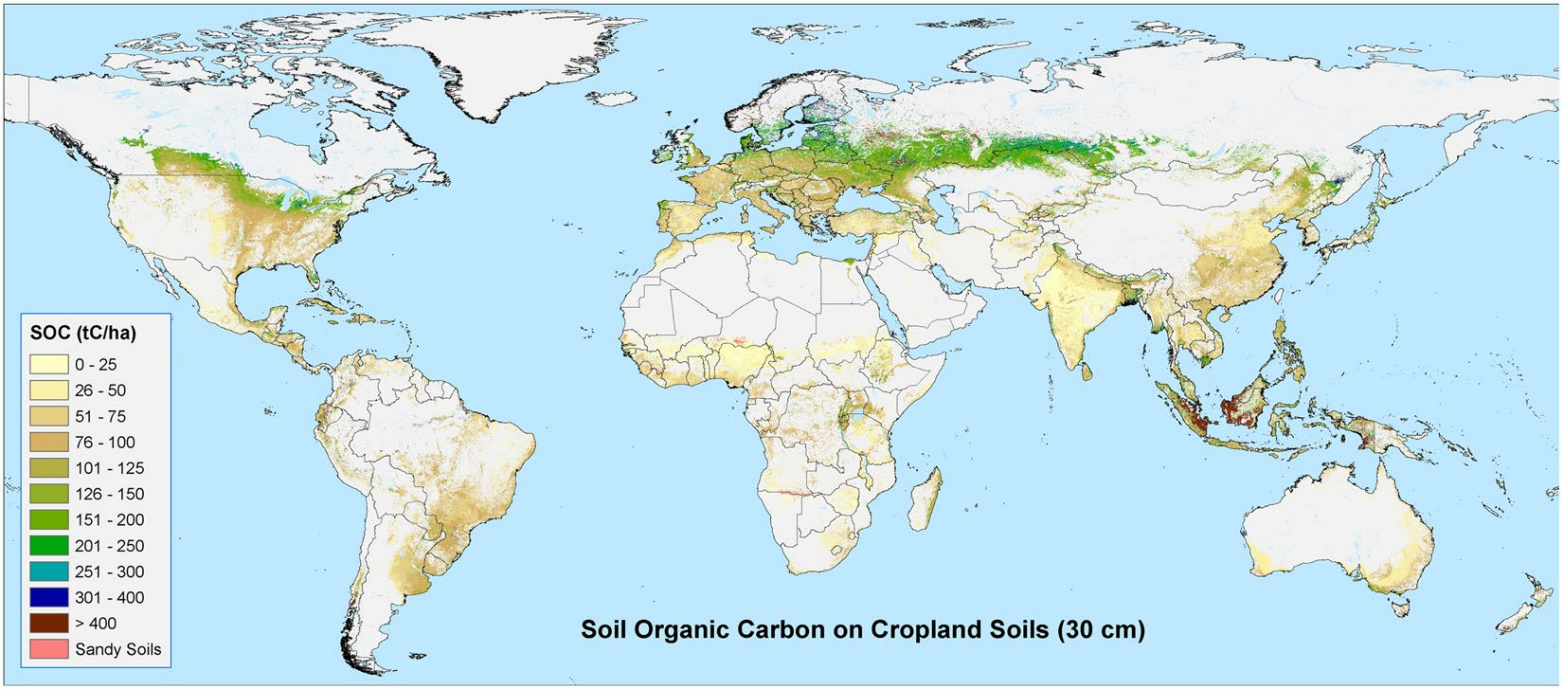
Soil organic carbon (SOC) in the top 30 cm, currently (T_0_), on all available cropland soils globally (i.e. those not excluded from the analysis as high SOC soils or sandy soils). Maps were produced based upon a geospatial analysis of datasets from the SoilsGrids250 database^19^, using ESRI ArcGIS software (version 10.3; www.esri.com).

Estimates of global soil carbon stocks, trends and sequestration potential^11,16^, particularly within the context of a warming climate^7,8,21,22^, are now central to important discussions ongoing within various international fora, notably the discussions on including agricultural land within mitigation strategies and protocols at the UNFCCC, and are the basis for the 4p1000 Initiative^14^. The spatial distribution of SOC on croplands (Fig. 1), and its contribution to total carbon stock, varies with latitude, and differs substantially from that of carbon stored in above and below ground biomass^23,24^. Most of the world’s SOC is stored at northern latitudes, particularly in the permafrost and moist boreal regions. In contrast, large areas of cropland in India, across the Sahel, northern China, and Australia are found on low carbon density soils. An overview of 27 studies^25^ reports that 1500 Pg C can be regarded as a rough estimate of the global SOC pool (to one meter depth; across all the world’s soils, more than 130 million km^2^), however with substantial variability among both spatially- and non-spatially-explicit estimates and a range of from 500 to 3000 Pg C.

About 372,000 km^2^ of cropland (Supplementary Figure S1), comprised of carbon dense soils (> 400 t C/ha and/or with a bulk density <1.0 g/cm^3^) and which are considered likely to lose SOC under any form of cropping management, and sandy soils unlikely to sequester carbon due to high sand content (> 85%), were excluded from the analysis as “unavailable” (Table 1). In particular, it is highlighted that high SOC soils, while accounting for only 2% of total cropland area, account for almost 6% (8.48 Pg C) of total global cropland SOC stocks, and require a set of management options aimed toward conservation and maintenance of carbon stocks^25^. These areas are primarily peatlands in South East Asia, Russia, some in North America, South America, Europe, Australia/Pacific, and Andosols in South America. Cultivation of peat soils has been shown to contribute significantly to global emissions from agriculture^26^. Tropical and temperate peatlands account for a disproportionate share of terrestrial carbon stocks considering their more limited area globally^27^, with peatland drainage, concentrated in Europe and Indonesia, reported to account for nearly a third of all cropland emissions^28^.

**Table 1:** Soil organic carbon (SOC) for all available cropland soils globally (i.e. those not excluded from the analysis as high SOC or sandy soils), showing both the global totals and the global averages per hectare, at current status (T_0_), and after 20 years for both the medium and high sequestration scenarios, and their annual increment.

**Cropland Soils** (30 cm depth)	**Soil Organic Carbon**					**Average Soil Organic Carbon per Hectare**					**Cropland Area**
	**Current**	**After 20 Years**		**Annual Increase**		**Current**	**After 20 Years**		**Annual Increase**		
**Scenario**	**T**_**0**_	**Medium**	**High**	**Medium**	**High**	**T**_**0**_	**Medium**	**High**	**Medium**	**High**	
	**PgC**			**PgC/yr**		**tC/ha**			**tC/ha/yr**		**km**^2^
**Available Soils**	131.81	149.84	168.87	0.90	1.85	82	93	105	0.56	1.15	15,935,304
**High SOC Soils**	8.34					252					331,227
**Sandy Soils**	0.14					34					40,999
**Total**	140.28										16,307,531

Globally, cropland stores more than 140 Pg C in the top 30 cm of soil, almost 10% of the total global SOC pool. About 94% of this carbon (131.81 Pg C) is stored on the 15.9 million km^2^ (98% of global cropland) identified as potentially available for enhanced carbon sequestration through improved soil management and farming practices^11^. Global distribution of SOC is strongly influenced by temperature and precipitation^15,29^. SOC is generally lower in the tropics where it is hotter and/or drier, and higher in the cooler, wetter, more northerly, and to a somewhat lesser extent, southerly, latitudes (Fig. 1). Lal (2002) cites several studies showing an exponential decrease in SOC with increase in temperature. This is reflected by low SOC values found across much of the equatorial belt (e.g. less than 100 t C/ha), with the highest carbon density soils (400 t C/ha or more) found in the northern croplands and farmed peat soils of the United States, Canada, Europe and Russia (see Supplementary Table S1).

The regions of North America, Eurasia (Russia) and Europe currently store the greatest amount of carbon on cropland, each with more than 21 Pg C, and all together accounting for over 50% of all SOC stocks on cropland globally (Table 1). By contrast, Central America, North Africa, and the Australian/Pacific region have very low amounts of stored SOC, together comprising 6.48 Pg C or just over 4.6% of the global total. Western Asia, South Asia, Southeast East Asia and East Asia each have moderate amount ranging from 4.38 Pg C to 9.14 Pg C, but together accounting for just less than 2% of global total. South America, even having a fairly large amount of farmland, has a moderate 9.42 Pg C. Almost 12 Pg C, more than 8.5% of the global total, is found in Africa, with the highest concentrations found in the Eastern and Central regions. Nationally, Russia with its vast northern tracts of carbon dense agricultural land has the largest total amount of SOC stored on cropland more than 21.9 Pg C (almost 17% of the global total), followed by the United States (18.9 Pg C), China (8.4 Pg C), India (6.4 Pg C), and Brazil (5.0 Pg C) (Supplementary Table S2).

### Sequestration Potential of Increased Soil Organic Carbon Stocks on Croplands

**Figure 2:**
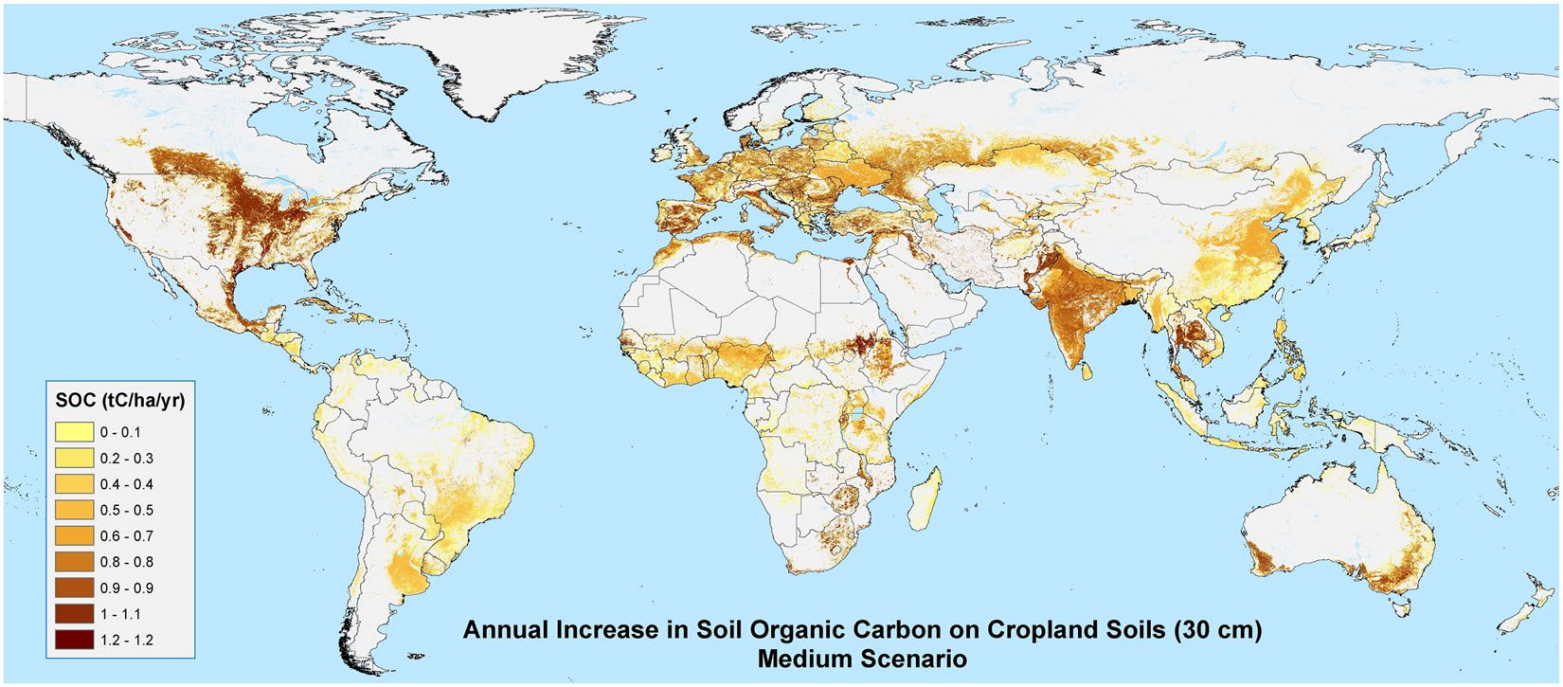
Annual increase in soil organic carbon (SOC) in the top 30 cm, on all available cropland soils globally (i.e. those not excluded from the analysis as high SOC or sandy soils) under the medium scenario (i.e. an increase in percent SOC of 0.27 over 20 years). Maps were produced based upon a geospatial analysis of datasets from the SoilsGrids250 database^19^, using ESRI ArcGIS software (version 10.3; www.esri.com).

Our analysis shows that if the SOC content in the 0–30 cm depth layer of all available cropland increased from 0.27% to 0.54%, i.e. from the Sommer and Bossio (2014) medium to high scenarios, from 0.56 to 1.15 t C/ha/yr could be sequestered (Table 1), which translates to 0.90 to 1.85 Pg C/yr globally, for at least the 20 years that sequestration can be expected to continue. This estimate of total sequestration potential on cropland soils compares well to and is in the range of other recent estimates for agricultural areas^5,6,9,12,17^. The annual increment (on a per hectare basis) ranged from 0.50 up to 1.28 t C/ha across the various regions (see Supplementary Figures S2–S9), however total amounts of sequestered carbon vary widely, due to the varying amount of cropland across regions. North America showed the highest potential for total carbon storage, globally, with between 0.17 and 0.35 Pg C (0.60 to 1.22 t/ha) sequestered annually (Table 2; Fig. 2). South Asia and Europe showed approximately the same total sequestration potential (0.11–0.23 Pg C/yr), second highest among regions, even though European soils are already fairly carbon dense on average (106 t C/ha). Low levels of SOC on croplands across South Asia (44 t C/ha), much of it with serious degradation issues, provide the opportunity to sequester 0.62–1.28 t C/ha/yr on over 2.9 million km^2^ of land, accounting for 2.2 to 4.5 Pg C/yr of carbon storage. Likewise, Africa, taken altogether with over 2.6 million km^2^ of cropland, shows a large potential for soil carbon storage, ranging from 0.15 to 0.31 Pg C/yr. South Asia and North Africa show the highest potential for carbon storage on a per hectare basis (0.62–1.28 t C/ha), although North Africa has very little cropland and hence only a small potential for total carbon soil sequestration.

**Table 2:** Regional Analysis of Available Soils: Soil organic carbon (SOC) for all available cropland soils by region (i.e. those not excluded from the analysis as high SOC or sandy soils), showing both the regional totals and the regional averages per hectare, at current status (T_0_), and after 20 years for both the medium and high sequestration scenarios, and their annual increment.

**Cropland Soils** (30 cm depth)	**Soil Organic Carbon**					**Average Soil Organic Carbon**					**Cropland Area**
	**Current**	**After 20 Years**		**Annual Increase**		**Current**	**After 20 Years**		**Annual Increase**		
**Scenario**	**T**_**0**_	**Medium**	**High**	**Medium**	**High**	**T**_**0**_	**Medium**	**High**	**Medium**	**High**	
**Region**	**PgC**			**PgC/yr**		**tC/ha**			**tC/ha/yr**		**km**^2^
**Australian/Pacific**	3.75	4.50	5.29	0.04	0.08	57	68	80	0.57	1.16	659,834
**Central America**	1.22	1.37	1.52	0.01	0.02	87	98	109	0.53	1.09	139,742
**Central Asia**	5.01	5.40	5.81	0.02	0.04	136	146	158	0.53	1.08	369,061
**East Asia**	9.14	10.52	11.97	0.07	0.14	72	83	95	0.54	1.12	1,262,134
**Eastern and Southern Africa**	5.64	6.80	8.02	0.06	0.12	53	64	76	0.55	1.13	1,055,461
**Europe**	21.05	23.26	25.60	0.11	0.23	106	117	128	0.55	1.14	1,995,564
**North Africa**	1.51	1.83	2.17	0.02	0.03	58	71	84	0.63	1.28	258,602
**North America**	28.07	31.52	35.16	0.17	0.35	97	109	122	0.60	1.22	1,260,786
**Russia**	21.94	23.19	24.52	0.06	0.13	174	184	194	0.50	1.02	1,235,827
**South America**	9.42	10.72	12.09	0.06	0.13	76	87	98	0.53	1.08	1,753,039
**South Asia**	7.68	9.87	12.18	0.11	0.22	44	56	69	0.62	1.28	852,619
**SouthEast Asia**	8.15	9.06	10.03	0.05	0.09	96	106	118	0.53	1.10	1,307,174
**West and Central Africa**	4.83	6.35	7.95	0.08	0.16	37	49	61	0.58	1.19	891,532
**Western Asia**	4.38	5.44	6.57	0.05	0.11	49	61	74	0.60	1.23	2,893,928
**Global**	**131.81**	**149.84**	**168.87**	**0.90**	**1.85**	**81.61**	**92.82**	**104.66**	**0.56**	**1.15**	**15,935,304**

Among countries of the world, the United States showed the highest total annual potential in SOC (1.2–2.6 Pg C), with an average increase of 0.62–1.27 t C/ha/yr on over 2 million km^2^ of cropland. However, India, with about the same high average increase per hectare (0.63–1.27 t C/ha/yr) on over 1.6 million km^2^ of cropland, shows a potential total annual increment ranging from 1.0 to over 2.1 Pg C. Likewise, China and Russia, each with about 1.2 million km^2^ of cropland, both have a large potential to increase total carbon storage, ranging from 0.63 to over 1.3 Pg C annually each. Many smaller countries exhibit high per hectare annual increments, although their total C sequestration is low due to small areas of cropland. For example, 45 countries are in the higher range above 0.58 to 1.2 t C/ha, however, many of these are found in drier climates where water and biomass availability may be a limiting factor.

## Discussion

### The Role of Soil Organic Carbon Stocks on Cropland

Increasing soil organic carbon on the vast areas of cropland globally which are already intensively managed^12^ is more immediately practical and likely than on the other available landuse types, e.g. forestry or grazing land. On these croplands adoption of improved management practices offers the opportunity to sequester significant amounts of carbon in the near term, and potentially to make an important contribution to global mitigation efforts. The 4p1000 Initiative has identified an aspirational sequestration target of 3.5 Pg C/yr to provide substantive global mitigation. Our estimates suggest that from 26% up to 53% (0.90–1.85 Pg C) of this target could be reached in the top 30 cm of cropland soils alone, and continue over at least 20 years after adoption of SOC enhancing management, such as incorporation of organic manures, cover cropping, mulching, conservation tillage, some types for agroforestry practices, rotational grazing, or other practices known to increase soil carbon at the decadal scale. This requires that croplands increase SOC storage between 0.55–1.15 t/ha/yr. However, as Sommer and Bossio (2014) point out, adoption can be expected to be phased in over a period of years, with delays in roll out as various countries, production systems and farm types may be slower to adopt improved practices. Likewise, our estimates do not account for differences in climate and important soil process issues, notably nutrient and water limitations, biomass production and turnover rates. However, given the large amount of cropland potentially available, sequestering carbon via increases in the soil component on agricultural land is an achievable and potentially effective route to quickly increasing CO_2_ sequestration in the near term. For comparison, above-ground losses due to tropical land use conversion are currently estimated at 0.6–1.2 Pg C yr-1^30^. A strategy of enhancing agriculture with soil carbon enriching improved practices, e.g. via appropriate policy mechanisms, thus offers significant potential to mitigate landuse related carbon emissions and provide an opportunity for agricultural production to positively contribute to global mitigation efforts. SOC may be either enhanced by, or enhance above- and below-ground biomass carbon on agricultural land^24^, allowing for synergistic increases in on-farm carbon stocks. Agroforestry systems and planting trees, for example, may increase soil carbon sequestration^12^.

### Multiple Benefits from Enhanced Soil Organic Carbon Stocks on Cropland

The benefits of increasing soil organic matter in croplands goes far beyond climate change mitigation potential. Facilitation of increased SOC through improved farming and soil conservation practices, enhancing resilience through improved fertility status and water holding capacity, also provide important adaptation benefits^31^. It is generally recognized that changes in the moisture regime (e.g. drought or heavy precipitation events) can significantly impact crop productivity^32^. These climatic conditions are mitigated by SOC^9,17,33^, which adds structure, improves water infiltration and holding capacity, increases cation exchange capacity, and impacts soil fertility, a major controlling factor of agricultural productivity and both regional and household food security^34^. Soil conditions have dramatic effects on the abundance and efficiency of N-fixing bacteria^35^, which are vitally important in cropping systems that lack fertilizer inputs^36^. Thus increased SOC through improved management practices is likely to add substantial resilience to croplands and farming systems, particularly during drought years or increased seasonal variability, helping to avoid edaphic (soil related) droughts that result from land degradation^37^.

For the most part, agricultural practices that increase soil organic matter are supportive of enhanced food production and other ecosystem services. This is in contrast to other proposed negative emission strategies, such as afforestation (plantations of fast growing trees) and BECCS (bioenergy and carbon capture and storage) that will entail destruction of huge amounts of natural ecosystems or productive agriculture land if implemented at scales large enough to impact CO_2_ in the atmosphere^38–40^. Given that hundreds of millions of small farmers for their subsistence depend upon croplands around the world, mitigation benefits of enhanced SOC storage must be recognized as only one significant component of an array of multiple benefits to achieve.

Despite the large technical potential to sequester carbon in soils, there are often significant limitations to achieving that potential in any particular place and within specific farming systems, including lack of biomass and other inputs^16^. In addition, there may be tradeoffs with productivity, food security or hydrologic balances^34,41^, as well as concerns regarding other GHGs, such as N_2_O^16^. As with any efforts to sustain notable changes in practice significant understanding of cultural, political and socioeconomic contexts are required^11^. Enhanced understanding of land potential is also necessary to target limited resources^37^. While numerous constraints to achieving the technical potential of soil carbon sequestration exist, there are cases in which far higher sequestration rates are proposed than commonly accepted, even for annual cropping systems^42^. Refining our understanding of the technical potential for carbon sequestration in soils, and the practical implementation of improved soil management and farming practices aimed towards increasing SOC, offers a strategy for mitigation within the land use sector in the near-term, with potentially positive implications for food security and ecological resilience in the long-term.

## Methods

Three publicly available global geospatial datasets were used for this study:

### Soils Data

The recently released Soils Grid 250 m is a global database of soil properties at a spatial resolution of 250 m, provided by ISRIC World Soil Information. Datasets for SOC (g/kg), bulk density (kg/m^3^) and sand content (weight %), at several depths and depth layers, from 0–30 cm, were utilized in the analysis^19^.

### Landcover Data

The Global Land Cover SHARE Beta-Release v1.0 (GLC_SHARE) fractional landcover geospatial database, provides an estimate of the percent of cropland area within a 1 km grid cell, and was used to identify cropland extent. The designation “cropland” is an aggregated category based upon the UN Land Cover Classification System, which includes herbaceous crops, woody crops, and multiple or layered crops. This dataset was resampled to allow for the analysis and geoprocessing at the finer 250 m (0.002083333 degree) resolution of the soils data^20^.

### Administrative Boundaries

The GADM database of Global Administrative Areas v 2.8 was used to analyze results by regions and countries^43^.

**Figure 3:**
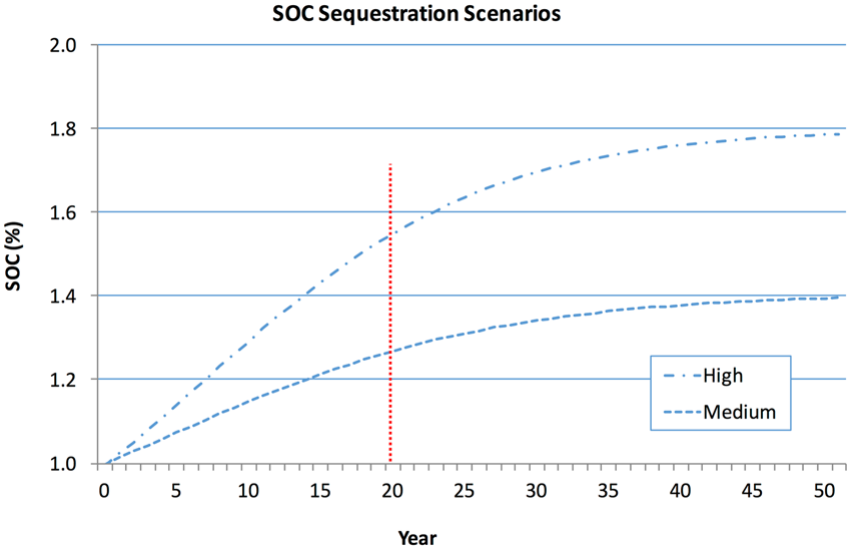
Increase in SOC (%) over time under two cropland sequestration scenarios (Eq. (1)). SOC_0_ in the shown cases is 0.60% and 0.71% for the high and medium graphs, respectively; note that the values for SOC_0_ are exemplary only and do not affect SOC sequestration rates. If the SOC content in the 0–30 cm depth layer of all available cropland increased 0.27% (medium scenario) to 0.54% (high scenario), then from 0.56 to 1.15 t C/ha/yr would be sequestered (Table 1), which translates to 0.90 to 1.85 Pg C/yr globally, for at least the minimum of 20 years that sequestration can be expected to continue.

The geospatial analysis uses the equation for potential carbon sequestration delineated in Sommer and Bossio (2014) as the estimate of the potential attainable increase of SOC on croplands after twenty years. These starting estimates, i.e. 0.27 increase for the medium scenario and 0.54 increase for the high scenario, are used to determine the estimate of SOC sequestration potential in tons of carbon (or Pg C), on a grid cell by grid cell basis. The SoilsGrid250m SOC and bulk density datasets were used to parameterize the conversion from SOC as a percent of the top 30 cm soil depth layer, to SOC in t C (or PgC for the global results). Only the medium and high sequestration scenarios pertinent to croplands are presented, since the low scenario in Sommer and Bossio (2014) refers to sequestration rates for unimproved pasture land. SOC sequestration. We have estimated the increase after a 20-year time span in order to provide an operational description of the linear portion of the SOC sequestration curve, noting that the rate of increase is likely to decrease sometime thereafter and eventually reach a new equilibrium (Fig. 3).

The increase in %-SOC in response to improved management or other sequestration measures was described in Sommer and Bosio (2014) with a four-parameter sigmoid function of the form:

1 $${\rm{S}}{\rm{O}}{\rm{C}}={{\rm{S}}{\rm{O}}{\rm{C}}}_{0}+\frac{a}{1+{e}^{-\frac{t-{t}_{0}}{b}}}$$

where *SOC*_0_ is the initial soil organic carbon content (%), *a* and *b* are empirical constants and *t* the time expressed in years. *t*_0_ is the year where the slope of the curve is largest, i.e. the annual sequestration rate highest. The parameters for the two scenarios (based upon Sommer and Bossio 2014), were:

$$\begin{array}{l}{\rm{Medium}}:\quad {{\rm{SOC}}}_{0}=0.71;\quad a=0.697;\quad b=11.5;\quad {t}_{0}=4\\ {\rm{High}}:\quad \quad \,{{\rm{SOC}}}_{0}=0.60;\quad a=1.20;\quad b=9.8;\quad {t}_{0}=7\end{array}$$

The percent increase of SOC after 20 years (T_20_) was calculated from this curve for the two scenarios as:

$$\begin{array}{l}{\rm{Medium}}:\quad {{\rm{T}}}_{20}=0.269663\\ {\rm{High}}:\quad \quad \,\,{{\rm{T}}}_{20}=0.553936\end{array}$$

Bulk density was used to first convert SOC (t/ha) (as presented in the SoilsGrid250m data) into SOC (%). The estimated percentage increase was then added, and the result was then converted back in SOC (t/ha).

High SOC soils were mapped in a separate layer by identifying grid cells with a weighted average bulk density (0–30 cm) equal to or less than 1.0 kg/m^3^, and/or any gird cells with more than 400 t C/ha. These high carbon soils were excluded from further analysis. Sandy soils were mapped in a separate layer by identifying grid cells with sand content (at 15 cm) equal to or greater than 85%, and likewise, were excluded from further analysis.

All result grids converted to World Sinusiodal projection to allow for area calculations. The GLCShare – Dominant (Class 2 = Cropland) dataset, in percent area of a 1 km grid cell, was resampled to 250 m and multiplied times the various results (t/ha) to calculate actual total tC in each grid cell, i.e. given the actual area of agricultural land in that grid cell. The sum of grid cells was then multiplied by the number of hectares to provide regional and country zonal statistics. A full description of the geospatial methodology used to calculate the agricultural area, percent SOC, the conversion to tons per hectare, and the aggregation of tons of SOC on cropland is given in the Supplemental Materials. Results datasets are archived and available from Harvard Dataverse, accessible from the following link: DOI: 10.7910/DVN/HYFICT.  High resolution maps are available online at: http://ciat.cgiar.org/global-soil-carbon

## Electronic supplementary material

Supplementary Information
