# Author Correction: Global Sequestration Potential of Increased Organic Carbon in Cropland Soils

**DOI:** 10.1038/s41598-021-98375-0

**Published:** 2021-09-15

**Authors:** Robert J. Zomer, Deborah A. Bossio, Rolf Sommer, Louis V. Verchot

**Affiliations:** 1 Key Laboratory for Plant Diversity and Biogeography of East Asia (KLPB), Kunming Institute of Botany, Chinese Academy of Science, Kunming, 650201 Yunnan China; 2 The Nature Conservatory (TNC), Santa Cruz, California USA; 3 International Center for Tropical Agriculture (CIAT), Nairobi, Kenya; 4 International Center for Tropical Agriculture (CIAT), Cali, Colombia

Correction to: *Scientific Reports* 10.1038/s41598-017-15794-8, published online 14 November 2017

The Article contains errors in Table 2, where the cropland area given in the last column ‘Cropland Area’ is incorrect for the regions North America, Russia. South America, South Asia, SouthEast Asia, West and Central Africa and Western Asia, stated in the first column ‘Cropland Soils (30cm depth)’. The correct Table 2 and accompanying legend appear below.

**Table 2:** Regional Analysis of Available Soils: Soil organic carbon (SOC) for all available cropland soils by region (i.e. those not excluded from the analysis as high SOC or sandy soils), showing both the regional totals and the regional averages per hectare, at current status (T_0_), and after 20 years for both the medium and high sequestration scenarios, and their annual increment.

Cropland Soils (30 cm depth)	Total Soil Organic Carbon					Average Soil Organic Carbon					Cropland Area
	Current	After 20 Years		Annual Increase		Current	After 20 Years		Annual Increase		
Scenario	T_0_	Medium	High	Medium	High	T_0_	Medium	High	Medium	High	
Region	PgC			PgC/yr		tC/ha			tC/ha/yr		(km^2^)
Australian/Pacific	3.75	4.50	5.29	0.04	0.08	57	68	80	0.57	1.16	659,834
Central America	1.22	1.37	1.52	0.01	0.02	87	98	109	0.53	1.09	139,742
Central Asia	5.01	5.40	5.81	0.02	0.04	136	146	158	0.53	1.08	369,061
East Asia	9.14	10.52	11.97	0.07	0.14	72	83	95	0.54	1.12	1,262,134
Eastern and Southern Africa	5.64	6.80	8.02	0.06	0.12	53	64	76	0.55	1.13	1,055,461
Europe	21.05	23.26	25.60	0.11	0.23	106	117	128	0.55	1.14	1,995,564
North Africa	1.51	1.83	2.17	0.02	0.03	58	71	84	0.63	1.28	258,602
North America	28.07	31.52	35.16	0.17	0.35	97	109	122	0.60	1.22	2,893,928
Russia	21.94	23.19	24.52	0.06	0.13	174	184	194	0.50	1.02	1,260,786
South America	9.42	10.72	12.09	0.06	0.13	76	87	98	0.53	1.08	1,235,827
South Asia	7.68	9.87	12.18	0.11	0.22	44	56	69	0.62	1.28	1,753,039
SouthEast Asia	8.15	9.06	10.03	0.05	0.09	96	106	118	0.53	1.10	852,619
West and Central Africa	4.83	6.35	7.95	0.08	0.16	37	49	61	0.58	1.19	1,307,174
Western Asia	4.38	5.44	6.57	0.05	0.11	49	61	74	0.60	1.23	891,532
Global	131.81	149.84	168.87	0.90	1.85	81.61	92.82	104.66	0.56	1.15	15,935,304

