# Supplementary material for: Global Sequestration Potential of Increased Organic Carbon in Cropland Soils
Source: Sci Rep. 2017 Nov 14;7:15554. doi: 10.1038/s41598-017-15794-8 (PMC5686149; doi:10.1038/s41598-017-15794-8)
Supplement: Supplementary file 1 — Supplementary Information [file 41598_2017_15794_MOESM1_ESM.pdf]

Global Sequestration Potential of Increased Organic Carbon in Cropland Soils

Supplementary Materials

October 21, 2017

Submitted to:

Scientific Reports

Authors:

Robert J. Zomer [1]*, Deborah Bossio[2], Rolf Sommer[3], Lou Verchot[4]

1. *Key Laboratory for Plant Diversity and Biogeography of East Asia (KLPB), Kunming Institute of Botany, Chinese Academy of Science, Kunming 650201, Yunnan, China Email: r.zomer@cgiar.org*
2. *The Nature Conservatory (TNC), Santa Cruz, California*
3. *International Center for Tropical Agriculture (CIAT), Soils Division, Nairobi, Kenya*
4. *International Center for Tropical Agriculture (CIAT), Cali, Colombia*

* Corresponding Author

Table of Contents

Methods:...3
Data sources:...4
Soils Data: SoilGrids250m ...4
Landcover Data: Global Land Cover-SHARE - Beta-Release 1.0 (Year 2014)...4
Administrative Boundaries: GADM ...5
Data Processing Steps: ...6
Calculating Increase at 20 years: ...6
Calculating tons of carbon (t C or Pg C) per region/country: ...7
Supplementary Tables:...8
Table S1: Regional Analysis of High SOC Soils: ...8
Table S2: National Analysis of SOC on Available Cropland Soils: ...9
Table S3: Top fifty countries with the highest total SOC on croplands, currently...13
Table S4: Top fifty countries with the highest total annual increment of SOC increase. ...14
Table S5: Top fifty countries with the highest total annual increment of SOC increase per hectare. .15
Table S6: Top forty countries with the highest total area of high SOC soils. ...16
Supplementary Figures:...17
Figure S1: High soil organic carbon (SOC) and sandy soils. ...17
Figure S2: Annual increase in soil organic carbon (SOC) – Africa ...18
Figure S3: Annual increase in soil organic carbon (SOC) – South America...19
Figure S4: Annual increase in soil organic carbon (SOC) – South Asia. ...20
Figure S5: Annual increase in soil organic carbon (SOC) – Southeast Asia...21
Figure S6: Annual increase in soil organic carbon (SOC) – East Asia. ...22
Figure S7: Annual increase in soil organic carbon (SOC) – Europe. ...23
Figure S8: Annual increase in soil organic carbon (SOC) – North America...24
Figure S9: Annual increase in soil organic carbon (SOC) – Australia ...25
References ...26

Methods:

The geospatial modeling and analysis uses the equation for potential carbon sequestration delineated in Sommer and Bossio (2014) as the estimate of the potential attainable increase of SOC on croplands after twenty years[1]. These starting estimates, i.e. 0.27 increase for the medium scenario and 0.54 increase for the high scenario, are used to determine the estimate of SOC sequestration potential in tons of carbon (or Pg C), on a grid cell by grid cell basis. The SoilsGrid250m SOC and bulk density datasets were used to parameterize the conversion from SOC as a percent of the top 30cm soil depth layer, to SOC in t C (or PgC for the global results). Only the medium and high sequestration scenarios pertinent to croplands are presented, since the low scenario in Sommer and Bossio (2014) refers to sequestration rates for unimproved pasture land. SOC sequestration[1]. We have estimated the increase after a 20-year time span in order to provide an operational description of the linear portion of the SOC sequestration curve, noting that the rate of increase is likely to decrease sometime thereafter and eventually reach a new equilibrium (Figure 3).

The increase in %-SOC in response to improved management or other sequestration measures was described in Sommer and Bossio (2014) with a four-parameter sigmoid function of the form[1]:

$$\mathrm{SOC} = \mathrm{SOC}_0 + \frac{a}{1+e^{-\frac{t-t_0}{b}}} \qquad (1)$$

where SOC_0 is the initial soil organic carbon content (%), a and b are empirical constants and t the time expressed in years. t_0 is the year where the slope of the curve is largest, i.e. the annual sequestration rate highest. The parameters for the two scenarios (based upon Sommer and Bossio 2014), were:

Medium: SOC_0 = 0.71; a = 0.697; b = 11.5; t_0 = 4

High: SOC_0 = 0.60; a = 1.20; b = 9.8; t_0 = 7

The percent increase of SOC after 20 years (T_{20}) was calculated from this curve for the two scenarios as:

Medium: T_{20} = 0.269663

High: T_{20} = 0.553936

Bulk density was used to first convert SOC (t/ha) (as presented in the SoilsGrid250m data) into SOC (%). The estimated percentage increase was then added, and the result was then converted back in SOC (t/ha).

High SOC soils were mapped in a separate layer by identifying grid cells with a weighted average bulk density (0-30 cm) equal to or less than 1.0 kg/m^3, and /or any gird cells with more than 400 t C/ha. These high carbon soils were excluded from further analysis. Sandy

soils were mapped in a separate layer by identifying grid cells with sand content (at 15 cm) equal to or greater than 85%, and likewise, were excluded from further analysis.

All result grids converted to World Sinusiodal projection to allow for area calculations. The GLCShare – Dominant (Class 2 = Cropland) dataset, in percent area of a 1 km grid cell, was resampled to 250m and multiplied times the various results (t/ha) to calculate actual total tC in each grid cell, i.e. given the actual area of cropland in that grid cell. The sum of grid cells was then multiplied by the number of hectares to provide regional and country zonal statistics. A full description of the geospatial methodology used to calculate the cropland area, percent SOC, the conversion to tons per hectare, and the aggregation of tons of SOC on cropland is given in the Supplemental Materials. All results datasets, including high resolution maps, are available online at: http://ciat.cgiar.org/global-soil-carbon

Data sources:

Soils Data: SoilGrids250m

Data downloaded from: http://soilgrid.org

Layers:

Soil organic carbon stock in tonnes per ha

Depth_1: 0 - 5 cm
Depth_2: 5 - 15 cm
Depth_3: 15 – 30 cm

Bulk density (fine earth) in kg / m^3

Depth_2: 5 cm
Depth_3: 15 cm
Depth_4: 30 cm

Resolution = 250 m

Reference:

Hengl, T., Mendes de Jesus, J., Heuvelink, G. B.M., Ruiperez Gonzalez, M., Kilibarda, M. et al. (2017) SoilGrids250m: global gridded soil information based on Machine Learning. PLoS ONE 12(2): e0169748. doi:10.1371/journal.pone.0169748[2].

Landcover Data: Global Land Cover-SHARE - Beta-Release 1.0 (Year 2014)

Dataset Manual: http://www.fao.org/land-water/land/land-governance/land-resources-planning-toolbox/category/details/en/c/1036355/

Data downloaded from: http://www.fao.org/geonetwork/srv/en/main.home?uuid=ba4526fd-cdbf-4028-a1bd-5a559c4bff38

Layers: GlcShare_v10_Dominant (used in analysis)
Dominant Landcover Type within Grid Cell

GlcShare_v10_02
Percent of Grid Cell under Cropland

Resolution = 30 arc-seconds (~1 sq.km)

Reference:

Latham J, Cumani R, Rosati I, Bloise M (2014) FAO Global Land Cover (GLC-SHARE) Beta-Release 1.0 Database, Version 1.0 - 2014 (Land and Water Division, FAO, Rome)[3].

The "Croplands" designation within the GLC-SHARE is an aggregated category based upon the UN Land Cover Classification System (LCCS v1x), which includes: *Herbaceous Crops:* The class is composed of a main layer of cultivated herbaceous plants (graminoids or forbs). It includes herbaceous crops used for hay. All the non-perennial crops that do not last for more than two growing seasons and crops like sugar cane where the upper part of the plant is regularly harvested while the root system can remain for more than one year in the field are included in this class.

Woody Crops: The class is composed of a main layer of permanent crops (trees and/or shrub crops) and includes all types of orchards and plantations (fruit trees, coffee and tea plantation, oil palms, rubber plantation, Christmas trees etc.).

Multiple or Layered crops: This class combines different land cover situations:

Two layers of different crops (woody + herbaceous): A common case is the presence of one layer of woody crops (trees or shrubs) and another layer of herbaceous crop, such as for wheat fields with olive trees in the Mediterranean area and intense horticulture, oasis or typical coastal African agriculture were herbaceous fields are covered by palm trees, etc.

Presence of one important layer of natural vegetation (mainly trees) that cover one layer of cultivated crops: A typical example are coffee plantations shadowed by natural trees in the equatorial area of Africa.

The Global Land Cover SHARE Beta-Release v1.0 (GLC_SHARE) fractional landcover geospatial database (at 1 km resolution), developed as an up-to-date amalgamation of the "best available data" by FAO, identifies about 16.3 million km^2 of cropland globally, roughly corresponding to non-spatially collected statistics provided by FAO for "arable and permanently cropped" area for the year 2013 (FAOSTAT, 2016). This accounts for about a third of the total of 49 million km^2 reported by FAO for total global cropland area in 2013 (but which includes large areas of pasture and grasslands). The designation "cropland" is an aggregated category based upon the UN Land Cover Classification System (LCCS v1x), which includes herbaceous crops, woody crops, and multiple or layered crops. The 16 million km^2 of cropland globally identified by the GLC-Share Database is a fairly conservative estimate, e.g. compared to the 22 million km^2 of cropland area identified in the widely used GLC2000. The GLC-Share estimate of cropland which we use in our analysis is more likely to be restricted to just cropped area (i.e. not agroforestry, tree crops, mixed farming, pastures or other grazing areas), and most recent global landcover database available.

Administrative Boundaries: GADM

The GADM database of Global Administrative Areas v 2.8 was used to analyze results by regions and countries[4].

Data downloaded from: http://gadm.org

Data Processing Steps:

Geoprocessing resolution - all steps and results: 0.002083333 degree (250 m in World Sinsoidal projection)

All steps only process grid cells with a landcover type of “Cropland”

GlcShare_v10_Dominant: Value = 2

All geoprocessing is done separately for each of 3 soil depth layers, which are added together in the last steps to make up the 30 cm soil depth layer

High SOC soils, e.g. peat soils or Histosols, were excluded from the analysis by

a.) excluding grid cells with a weighted average bulk density (0-15 cm) of less than 1.0
b.) excluding grid cells with greater than 400 t/ha of SOC

Sandy soils. i.e. soils with >= 85% sand content at 15 cm depth, were likewise excluded

Calculating Increase at 20 years:

Percent increase after 20 years calculated from the curve in Sommers and Bossio (2016) for two scenarios (see associated Excel spreadsheet):

Medium: T_{20} = 0.269663

High: T_{20} = 0.553936

1.) Converting SOC t/ha to SOC (%)

soc_%_1 = (((soc_1/(soil_bd_1 * 10000 * depth_1)) * 100) * 1000)

Where:

soc_1 = soc (t/ha) at depth_1
soil_bd = soil bulk density at depth_1

2.) Adding percent increase at t_{20}

soc_%_t_{20}_1 = (soc_%_1 + t_{20})

Where:

soc_%_t_{20}_1 = soc (%) at t_{20}

3.) Converting SOC(%) at t_{20} to SOC t/ha

soc_t_{20}_1 = ((soc_%_t_{20}_1 /100)*(10000 * soil_bd_1 * depth_1)/1000)

Where:

soc_t_{20}_1 = soc (t/ha) at t_{20}

4.) Adding depth layers together to make 0-30 cm depth layer"

$soc\_ t_0\_30cm = (soc\_1 + soc\_2 + soc\_3)$

$soc\_t_{20}\_30cm = (soc\_t_{20}\_1 + soc\_t_{20}\_2 + soc\_t_{20}\_3)$

5.) Calculating increase in SOC at year 20

$soc\_diff\_30cm = (soc\_t_{20}\_30cm - soc\_ t_0\_30cm)$

6.) Identifying high carbon soils

High SOC soils, e.g. peat soils, Histosols, or Andosols, were mapped in a separate layer by identifying grid cells with a weighted average bulk density (0-30 cm) equal to or less than 1.0 g/cm^3, and /or any gird cells with more than 400 t C/ha. These high carbon soils were excluded from further analysis.

7.) Identifying sandy soils

Sandy soils were mapped in a separate layer by identifying grid cells with sand content (at 15 cm) equal to or greater than 85%. These high sand content soils were excluded from further analysis.

Calculating tons of carbon (t C or Pg C) per region/country:

8.) All result grids converted to World Sinusiodal projection to allow for area calculations

9.) GLCShare – Dominant (Class 2 = Cropland) in percent area of 1 km grid cell, resampled to 250m and multiplied times the various results (t/ha) to calculate actual total tC in each grid cell, i.e. given the area of cropland in that grid cell.

soc_t_x * GLCShare (% ag land in grid cell) = Actual tC per grid cell

10.) Sum of grid cells, multiplied 6.25 (the number of hectares in a 250 m grid cell), to provide regional and country zonal statistics.

Supplementary Tables:

Table S1: Regional Analysis of High SOC Soils:

Soil organic carbon (SOC) for all available cropland soils regionally (i.e. those not excluded from the analysis as high SOC or sandy soils), showing the regional totals and averages, at current status (T_0) and after 20 years (T_{20}), for the medium and high sequestration scenarios, and their annual increment.

**Agricultural Land**<br>**High SOC Soils**	**Total Soil Organic Carbon**<br>**T_0**	**Average per Hectare**<br>**T_0**	**Agricultural Area**
**Region**	PgC	t/ha	(km^2)
**Australian / Pacific**	0.32	189.3	16,918
**Central America**	0.01	167.1	694
**Central Asia**	0.08	379.5	2,195
**East Asia**	0.16	250.9	6,249
**Eastern and Southern Africa**	0.08	134.9	5,624
**Europe**	0.51	253.8	19,909
**North Africa**	0.00	354.3	1
**Russia**	2.01	278.6	72,202
**South America**	0.49	161.8	30,008
**South Asia**	0.05	255.3	1,939
**SouthEast Asia**	4.08	261.2	156,138
**West and Central Africa**	0.06	169.7	3,460
**Western Asia**	0.02	325.7	587
**North America**	0.48	313.2	15,304
**Global**	8.34	251.6	331,227

Table S2: National Analysis of SOC on Available Cropland Soils: Soil organic carbon (SOC) for all available cropland soils by country (i.e. those not excluded from the analysis as high SOC or sandy soils), showing the regional totals and averages, at current status (T_0) and after 20 years (T_{20}), for the medium and high sequestration scenarios, and their annual increment.

Available Soils	Total Soil Organic Carbon			Annual Increment		Average per Hectare			Annual Increment		Agricultural Area
Region	T_0	Medium T_{20}	High T_{20}	Medium	High	T_0	Medium T_{20}	High T_{20}	Medium	High	
Country	Megatons (t C * 10^6)					tC/ha					(km^2)
**Australia / Pacific**											
Australia	3,418	4,143	4,907	36.23	74.46	54	65	77	0.57	1.17	634,882
Fiji	16	17	19	0.07	0.15	106	116	126	0.49	1.01	1,472
New Caledonia	1	1	1	0.01	0.01	82	93	104	0.53	1.08	132
New Zealand	235	250	266	0.74	1.53	145	154	164	0.46	0.94	16,244
Papua New Guinea	72	78	84	0.28	0.58	119	128	138	0.47	0.96	6,054
Vanuatu	9	10	11	0.03	0.07	124	133	143	0.47	0.97	737
**Central Asia**											
Kazakhstan	4,179	4,444	4,723	13.24	27.21	161	171	182	0.51	1.05	260,033
Kyrgyzstan	184	204	225	1.00	2.05	102	113	125	0.56	1.14	17,953
Mongolia	235	253	273	0.92	1.90	128	139	149	0.50	1.04	18,281
Tajikistan	76	88	100	0.58	1.20	74	86	98	0.57	1.17	10,223
Uzbekistan	250	299	352	2.47	5.09	59	71	84	0.59	1.21	42,089
**Central America**											
Belize	11	13	14	0.08	0.16	81	92	103	0.55	1.13	1,397
Costa Rica	40	45	49	0.22	0.45	94	104	115	0.51	1.05	4,269
Cuba	383	429	478	2.29	4.71	89	99	110	0.53	1.09	43,258
Dominican Republic	102	114	127	0.60	1.23	95	107	118	0.56	1.15	10,708
El Salvador	48	56	65	0.41	0.83	62	72	83	0.52	1.07	7,823
Guatemala	181	201	223	1.03	2.11	89	99	110	0.51	1.04	20,205
Haiti	75	84	94	0.47	0.97	88	99	111	0.56	1.14	8,484
Honduras	120	134	149	0.70	1.44	90	101	112	0.53	1.08	13,283
Jamaica	20	22	24	0.09	0.19	118	129	140	0.54	1.10	1,684
Nicaragua	162	184	206	1.07	2.20	79	90	101	0.52	1.08	20,451
Panama	65	72	80	0.37	0.76	93	104	115	0.53	1.09	6,962
Puerto Rico	7	8	9	0.04	0.08	105	117	129	0.57	1.18	712
**East Asia**											
China	8,408	9,717	11,098	65.42	134.48	70	81	93	0.55	1.12	1,197,627
Japan	452	486	521	1.67	3.43	137	147	158	0.51	1.04	32,983
North Korea	169	187	207	0.92	1.89	97	107	118	0.53	1.08	17,446
South Korea	106	120	134	0.70	1.44	82	92	104	0.54	1.11	12,942
Taiwan	10	11	12	0.06	0.13	87	99	111	0.59	1.20	1,103
**Eastern and Southern Africa**											
Angola	147	184	222	1.81	3.72	47	58	71	0.58	1.19	31,399
Botswana	7	10	14	0.18	0.37	24	37	50	0.63	1.30	2,829
Burundi	125	138	151	0.65	1.33	100	111	122	0.52	1.07	12,441
Eritrea	14	24	34	0.47	0.96	18	30	42	0.59	1.21	7,954
Ethiopia	1,268	1,533	1,812	13.22	27.17	47	57	68	0.49	1.02	267,329
Kenya	414	468	525	2.70	5.55	79	89	100	0.51	1.05	52,702
Lesotho	32	38	44	0.30	0.62	59	70	82	0.56	1.15	5,402
Madagascar	265	294	324	1.42	2.92	92	102	113	0.49	1.02	28,732
Malawi	203	256	311	2.63	5.40	44	55	67	0.57	1.17	46,307
Mozambique	238	291	348	2.68	5.50	50	62	73	0.57	1.16	47,315
Namibia	12	19	26	0.34	0.70	22	34	47	0.62	1.27	5,488
Rwanda	153	167	182	0.70	1.43	112	122	133	0.51	1.04	13,723
Somalia	118	148	180	1.50	3.09	46	58	70	0.59	1.21	25,621
South Africa	817	999	1,191	9.09	18.69	51	63	75	0.57	1.17	159,545
Tanzania	726	891	1,065	8.25	16.95	50	62	74	0.57	1.17	144,322
Uganda	590	655	723	3.24	6.66	95	106	117	0.52	1.08	61,810
Zambia	143	177	213	1.71	3.51	49	61	73	0.59	1.21	29,111
Zimbabwe	143	177	213	1.71	3.51	49	61	73	0.59	1.21	29,111

Table S2: (Cont.): National Analysis of SOC on Available Cropland Soils: Soil organic carbon (SOC) for all available cropland soils by country (i.e. those not excluded from the analysis as high SOC soils or sandy soils), showing the regional totals and averages, at current status (T_0) and after 20 years (T_{20}), for the medium and high sequestration scenarios, and their annual increment.

**Available Soils**	**Total Soil Organic Carbon**			**Annual Increment**		**Average per Hectare**			**Annual Increment**		**Agricultural Area**
**Region**	**T_0**	**Medium T_{20}**	**High T_{20}**	**Medium**	**High**	**T_0**	**Medium T_{20}**	**High T_{20}**	**Medium**	**High**	
**Country**	Megatons (t C * 10^6)					tC/ha					(km^2)
**Eurasia (Russia)**											
Russia	21,943	23,195	24,516	62.59	128.65	174	184	194	0.50	1.02	1,260,811
**Europe**											
Albania	62	69	77	0.37	0.77	99	111	124	0.60	1.23	6,206
Austria	199	221	244	1.08	2.23	99	110	121	0.54	1.11	20,127
Belarus	927	985	1,045	2.86	5.87	171	181	193	0.53	1.08	54,268
Belgium	87	101	116	0.70	1.43	66	76	87	0.52	1.08	13,261
Bosnia and Herzegovina	152	168	185	0.81	1.66	107	118	130	0.57	1.16	14,225
Bulgaria	484	549	617	3.22	6.62	88	100	112	0.59	1.20	55,037
Croatia	176	197	221	1.10	2.25	92	104	116	0.58	1.18	19,038
Czech Republic	351	391	433	2.00	4.11	94	105	116	0.54	1.10	37,234
Denmark	376	400	425	1.19	2.45	163	174	185	0.52	1.06	22,989
Estonia	197	207	217	0.49	1.01	210	221	232	0.53	1.08	9,372
Finland	488	506	524	0.88	1.81	257	266	276	0.46	0.95	18,996
France	1,841	2,100	2,373	12.94	26.58	79	90	102	0.56	1.14	232,344
Germany	1,824	1,997	2,180	8.66	17.80	112	123	134	0.53	1.09	162,640
Greece	215	246	280	1.59	3.26	80	92	105	0.59	1.22	26,793
Hungary	410	471	535	3.03	6.24	75	86	98	0.56	1.14	54,528
Ireland	150	156	163	0.33	0.68	208	217	227	0.46	0.94	7,204
Italy	1,103	1,255	1,414	7.57	15.56	86	98	110	0.59	1.21	128,138
Kosovo	31	36	40	0.21	0.43	88	99	111	0.58	1.19	3,587
Latvia	333	350	367	0.84	1.72	209	220	231	0.53	1.08	15,931
Lithuania	614	651	691	1.87	3.85	179	190	202	0.55	1.13	34,204
Luxembourg	11	12	13	0.04	0.09	126	136	147	0.50	1.04	854
Macedonia	47	54	62	0.38	0.78	72	83	96	0.58	1.20	6,501
Moldova	204	226	250	1.11	2.29	97	108	119	0.53	1.09	20,978
Montenegro	24	26	28	0.08	0.17	150	161	172	0.52	1.06	1,624
Netherlands	152	163	174	0.53	1.10	141	151	161	0.49	1.01	10,793
Norway	71	74	77	0.15	0.31	216	225	235	0.47	0.96	3,273
Poland	1,762	1,937	2,121	8.75	17.99	110	121	132	0.55	1.12	160,230
Portugal	321	366	413	2.25	4.63	86	98	111	0.61	1.25	37,153
Romania	937	1,053	1,175	5.79	11.90	90	102	113	0.56	1.15	103,550
Serbia	360	408	459	2.40	4.93	86	97	109	0.57	1.17	42,015
Slovakia	163	182	201	0.91	1.87	96	107	118	0.54	1.10	16,991
Slovenia	65	70	76	0.27	0.55	129	140	151	0.54	1.10	5,002
Spain	1,371	1,650	1,943	13.93	28.62	60	72	85	0.61	1.25	229,705
Sweden	523	549	577	1.33	2.73	195	205	216	0.50	1.02	26,772
Switzerland	129	139	148	0.47	0.96	141	151	162	0.51	1.05	9,164
Ukraine	4,159	4,505	4,870	17.29	35.53	129	139	151	0.53	1.10	323,202
United Kingdom	732	793	858	3.07	6.30	119	129	140	0.50	1.03	61,457
**North Africa**											
Algeria	410	499	592	4.41	9.07	57	70	83	0.62	1.27	71,538
Egypt	408	457	508	2.42	4.98	105	118	131	0.62	1.28	38,877
Libya	66	92	120	1.30	2.68	32	44	57	0.62	1.28	20,867
Mauritania	6	9	13	0.18	0.36	20	32	45	0.62	1.27	2,857
Morocco	440	543	651	5.13	10.54	54	67	80	0.63	1.29	81,494
Tunisia	177	232	289	2.72	5.59	41	54	67	0.63	1.30	42,966
**North America**											
Canada	6,987	7,522	8,087	26.78	55.01	138	149	160	0.53	1.09	506,178
Mexico	2,137	2,559	3,004	21.10	43.35	57	68	80	0.56	1.15	377,829
United States	18,948	21,442	24,071	124.66	256.14	94	107	120	0.62	1.27	2,009,930

Table S2: (Cont.): National Analysis of SOC on Available Cropland Soils: Soil organic carbon (SOC) for all available cropland soils by country (i.e. those not excluded from the analysis as high SOC or sandy soils), showing the regional totals and averages, at current status (T_0) and after 20 years (T_{20}), for the medium and high sequestration scenarios, and their annual increment.

Available Soils	Total Soil Organic Carbon			Annual Increment		Average per Hectare			Annual Increment		Agricultural Area
Region	T_0	Medium T_{20}	High T_{20}	Medium	High	T_0	Medium T_{20}	High T_{20}	Medium	High	
Country	Megatons (t C * 10^6)					tC/ha					(km^2)
**South America**											
Argentina	2,199	2,523	2,865	16.20	33.29	70	81	92	0.52	1.07	312,239
Bolivia	266	307	350	2.03	4.18	72	83	95	0.55	1.13	36,986
Brazil	5,063	5,781	6,539	35.88	73.79	75	85	96	0.53	1.09	679,540
Chile	135	147	159	0.58	1.19	114	124	134	0.49	1.01	11,843
Colombia	301	333	366	1.57	3.23	99	110	120	0.52	1.06	30,359
Ecuador	198	214	232	0.83	1.70	111	120	130	0.46	0.95	17,842
French Guiana	1	1	2	0.01	0.01	90	100	110	0.49	1.00	147
Guyana	41	45	50	0.22	0.46	98	108	119	0.53	1.09	4,181
Paraguay	335	376	421	2.09	4.30	85	96	107	0.53	1.09	39,385
Peru	328	365	404	1.86	3.83	88	98	109	0.50	1.03	37,083
Suriname	6	7	7	0.03	0.06	116	126	137	0.53	1.08	533
Uruguay	331	371	413	2.00	4.10	95	106	118	0.57	1.17	35,027
Venezuela	211	245	280	1.68	3.45	70	81	93	0.55	1.14	30,289
**South Asia**											
Bangladesh	693	748	807	2.78	5.71	132	142	154	0.53	1.09	52,551
Bhutan	33	35	37	0.11	0.22	148	158	168	0.49	1.00	2,204
India	6,433	8,509	10,699	103.80	213.30	39	52	65	0.63	1.29	1,647,998
Nepal	326	361	397	1.73	3.55	100	111	122	0.53	1.09	32,560
Sri Lanka	197	216	237	0.96	1.97	111	122	133	0.54	1.11	17,740
**SouthEast Asia**											
Cambodia	408	462	520	2.74	5.62	87	99	111	0.59	1.20	46,691
East Timor	38	43	48	0.24	0.50	80	90	101	0.51	1.06	4,734
Indonesia	2,441	2,614	2,800	8.67	17.97	128	137	147	0.46	0.94	190,249
Laos	111	128	145	0.81	1.66	75	86	97	0.54	1.12	14,834
Malaysia	632	677	724	2.23	4.58	135	145	155	0.48	0.98	46,673
Myanmar	870	988	1,112	5.88	12.09	83	95	106	0.56	1.16	104,439
Philippines	851	936	1,027	4.29	8.83	104	114	125	0.52	1.08	81,878
Thailand	1,909	2,236	2,580	16.31	33.51	69	80	93	0.59	1.20	278,690
Vietnam	886	975	1,069	4.45	9.15	105	116	127	0.53	1.09	84,234
**West and Central Africa**											
Benin	80	111	143	1.53	3.14	29	41	52	0.56	1.15	27,320
Burkina Faso	82	144	209	3.08	6.34	16	29	42	0.62	1.27	49,942
Cameroon	327	396	469	3.45	7.10	52	63	74	0.55	1.12	63,147
Central African Republic	99	119	139	0.97	2.01	54	65	76	0.54	1.10	18,207
Chad	180	231	284	2.54	5.23	43	55	68	0.61	1.25	41,782
Coate d'Ivoire	330	403	479	3.62	7.44	52	63	75	0.57	1.17	63,560
D. R. Congo	351	391	433	2.00	4.11	94	105	116	0.54	1.10	37,234
Equatorial Guinea	21	23	25	0.10	0.20	102	112	123	0.50	1.02	2,004
Gabon	43	47	52	0.22	0.46	98	109	120	0.52	1.07	4,331
Gambia	14	18	22	0.20	0.42	42	55	68	0.63	1.30	3,229
Ghana	289	358	432	3.48	7.15	47	58	70	0.57	1.16	61,352
Guinea	175	210	247	1.76	3.63	50	60	71	0.51	1.04	34,791
Guinea-Bissau	35	41	47	0.29	0.60	61	72	82	0.51	1.05	5,726
Liberia	40	47	54	0.34	0.70	64	75	87	0.55	1.12	6,218
Mali	105	173	245	3.39	6.97	19	31	44	0.61	1.25	55,655
Niger	127	202	280	3.71	7.63	20	32	44	0.58	1.20	63,616
Nigeria	894	1,289	1,706	19.77	40.64	26	37	49	0.57	1.16	349,174
Republic of Congo	43	48	54	0.26	0.54	86	97	108	0.53	1.10	4,952
Senegal	137	186	238	2.45	5.03	35	47	60	0.62	1.27	39,540
Sierra Leone	98	114	131	0.80	1.64	64	75	86	0.52	1.08	15,243
South Sudan	145	176	208	1.52	3.12	53	64	76	0.55	1.14	27,450
Sudan	550	850	1,166	14.97	30.77	24	36	50	0.64	1.32	233,104
Togo	76	100	124	1.18	2.42	35	46	58	0.55	1.13	21,451

Table S2: (Cont.): National Analysis of SOC on Available Cropland Soils: Soil organic carbon (SOC) for all available cropland soils by country (i.e. those not excluded from the analysis as high SOC or sandy soils), showing the regional totals and averages, at current status (T_0) and after 20 years (T_{20}), for the medium and high sequestration scenarios, and their annual increment.

Available Soils	Total Soil Organic Carbon			Annual Increment		Average per Hectare			Annual Increment		Agricultural Area
Region	T_0	Medium T_{20}	High T_{20}	Medium	High	T_0	Medium T_{20}	High T_{20}	Medium	High	
Country	Megatons (t C * 10^6)					tC/ha					(km^2)
**Western Asia**											
Afghanistan	316	406	502	4.54	9.32	41	52	65	0.58	1.20	77,562
Armenia	55	61	67	0.27	0.56	109	120	131	0.54	1.10	5,092
Azerbaijan	175	197	221	1.12	2.30	89	100	112	0.57	1.17	19,661
Cyprus	12	15	18	0.15	0.32	50	63	76	0.64	1.31	2,421
Georgia	68	74	81	0.32	0.65	116	127	139	0.54	1.11	5,831
Iran	479	645	819	8.27	17.00	34	46	58	0.59	1.21	140,542
Iraq	239	324	414	4.26	8.75	34	47	60	0.61	1.26	69,555
Israel	21	26	31	0.25	0.51	53	66	79	0.63	1.30	3,952
Jordan	12	17	21	0.22	0.45	34	46	59	0.61	1.26	3,592
Lebanon	19	22	26	0.18	0.36	66	78	91	0.62	1.27	2,838
Pakistan	832	1,087	1,357	12.77	26.23	41	53	66	0.62	1.28	205,228
Palestine	4	5	6	0.04	0.08	61	74	87	0.63	1.30	643
Saudi Arabia	42	64	87	1.10	2.27	23	35	48	0.61	1.26	18,053
Syria	203	263	326	2.99	6.14	42	54	67	0.61	1.26	48,738
Turkey	1,855	2,178	2,519	16.15	33.19	67	79	91	0.59	1.20	275,553
Turkmenistan	90	115	141	1.23	2.53	44	56	69	0.60	1.23	20,479
Yemen	25	36	49	0.58	1.19	26	39	51	0.61	1.26	9,461

Table S3: Top fifty countries with the highest total SOC on croplands, currently.

Sorted by T0 - Total Soil Organic Carbon Currently

		Total Soil Organic Carbon			Annual Increment		Average per Hectare			Annual Increment		Agricultural Area
		T0	Medium T20	High T20	Medium	High	T0	Medium T20	High T20	Medium	High	
	Country	Megatons (t C * 106)					tC/ha					(km2)
1	Russia	21,943	23,195	24,516	62.59	128.65	174	184	194	0.50	1.02	1,260,811
2	United States	18,948	21,442	24,071	124.66	256.14	94	107	120	0.62	1.27	2,009,930
3	China	8,408	9,717	11,098	65.42	134.48	70	81	93	0.55	1.12	1,197,627
4	Canada	6,987	7,522	8,087	26.78	55.01	138	149	160	0.53	1.09	506,178
5	India	6,433	8,509	10,699	103.80	213.30	39	52	65	0.63	1.29	1,647,998
6	Brazil	5,063	5,781	6,539	35.88	73.79	75	85	96	0.53	1.09	679,540
7	Kazakhstan	4,179	4,444	4,723	13.24	27.21	161	171	182	0.51	1.05	260,033
8	Ukraine	4,159	4,505	4,870	17.29	35.53	129	139	151	0.53	1.10	323,202
9	Australia	3,418	4,143	4,907	36.23	74.46	54	65	77	0.57	1.17	634,882
10	Indonesia	2,441	2,614	2,800	8.67	17.97	128	137	147	0.46	0.94	190,249
11	Argentina	2,199	2,523	2,865	16.20	33.29	70	81	92	0.52	1.07	312,239
12	Mexico	2,137	2,559	3,004	21.10	43.35	57	68	80	0.56	1.15	377,829
13	Thailand	1,909	2,236	2,580	16.31	33.51	69	80	93	0.59	1.20	278,690
14	Turkey	1,855	2,178	2,519	16.15	33.19	67	79	91	0.59	1.20	275,553
15	France	1,841	2,100	2,373	12.94	26.58	79	90	102	0.56	1.14	232,344
16	Germany	1,824	1,997	2,180	8.66	17.80	112	123	134	0.53	1.09	162,640
17	Poland	1,762	1,937	2,121	8.75	17.99	110	121	132	0.55	1.12	160,230
18	Spain	1,371	1,650	1,943	13.93	28.62	60	72	85	0.61	1.25	229,705
19	Ethiopia	1,268	1,533	1,812	13.22	27.17	47	57	68	0.49	1.02	267,329
20	Italy	1,103	1,255	1,414	7.57	15.56	86	98	110	0.59	1.21	128,138
21	Romania	937	1,053	1,175	5.79	11.90	90	102	113	0.56	1.15	103,550
22	Belarus	927	985	1,045	2.86	5.87	171	181	193	0.53	1.08	54,268
23	Nigeria	894	1,289	1,706	19.77	40.64	26	37	49	0.57	1.16	349,174
24	Vietnam	886	975	1,069	4.45	9.15	105	116	127	0.53	1.09	84,234
25	Myanmar	870	988	1,112	5.88	12.09	83	95	106	0.56	1.16	104,439
26	Philippines	851	936	1,027	4.29	8.83	104	114	125	0.52	1.08	81,878
27	Pakistan	832	1,087	1,357	12.77	26.23	41	53	66	0.62	1.28	205,228
28	South Africa	817	999	1,191	9.09	18.69	51	63	75	0.57	1.17	159,545
29	United Kingdom	732	793	858	3.07	6.30	119	129	140	0.50	1.03	61,457
30	Tanzania	726	891	1,065	8.25	16.95	50	62	74	0.57	1.17	144,322
31	Bangladesh	693	748	807	2.78	5.71	132	142	154	0.53	1.09	52,551
32	Malaysia	632	677	724	2.23	4.58	135	145	155	0.48	0.98	46,673
33	Lithuania	614	651	691	1.87	3.85	179	190	202	0.55	1.13	34,204
34	Uganda	590	655	723	3.24	6.66	95	106	117	0.52	1.08	61,810
35	Sudan	550	850	1,166	14.97	30.77	24	36	50	0.64	1.32	233,104
36	Sweden	523	549	577	1.33	2.73	195	205	216	0.50	1.02	26,772
37	Finland	488	506	524	0.88	1.81	257	266	276	0.46	0.95	18,996
38	Bulgaria	484	549	617	3.22	6.62	88	100	112	0.59	1.20	55,037
39	Iran	479	645	819	8.27	17.00	34	46	58	0.59	1.21	140,542
40	Japan	452	486	521	1.67	3.43	137	147	158	0.51	1.04	32,983
41	Morocco	440	543	651	5.13	10.54	54	67	80	0.63	1.29	81,494
42	Kenya	414	468	525	2.70	5.55	79	89	100	0.51	1.05	52,702
43	Algeria	410	499	592	4.41	9.07	57	70	83	0.62	1.27	71,538
44	Hungary	410	471	535	3.03	6.24	75	86	98	0.56	1.14	54,528
45	Egypt	408	457	508	2.42	4.98	105	118	131	0.62	1.28	38,877
46	Cambodia	408	462	520	2.74	5.62	87	99	111	0.59	1.20	46,691
47	Cuba	383	429	478	2.29	4.71	89	99	110	0.53	1.09	43,258
48	Denmark	376	400	425	1.19	2.45	163	174	185	0.52	1.06	22,989
49	Serbia	360	408	459	2.40	4.93	86	97	109	0.57	1.17	42,015
50	Czech Republic	351	391	433	2.00	4.11	94	105	116	0.54	1.10	37,234

Table S4: Top fifty countries with the highest total annual increment of SOC increase.

	Total Soil Organic Carbon			Annual Increment		Average per Hectare			Annual Increment		Agricultural
	T0	Medium T20	High T20	Medium	High	T0	Medium T20	High T20	Medium	High	Area
Country	Megatons (t C * 106)					tC/ha					(km2)
United States	18,948	21,442	24,071	124.66	256.14	94	107	120	0.62	1.27	2,009,930
India	6,433	8,509	10,699	103.80	213.30	39	52	65	0.63	1.29	1,647,998
China	8,408	9,717	11,098	65.42	134.48	70	81	93	0.55	1.12	1,197,627
Russia	21,943	23,195	24,516	62.59	128.65	174	184	194	0.50	1.02	1,260,811
Australia	3,418	4,143	4,907	36.23	74.46	54	65	77	0.57	1.17	634,882
Brazil	5,063	5,781	6,539	35.88	73.79	75	85	96	0.53	1.09	679,540
Canada	6,987	7,522	8,087	26.78	55.01	138	149	160	0.53	1.09	506,178
Mexico	2,137	2,559	3,004	21.10	43.35	57	68	80	0.56	1.15	377,829
Nigeria	894	1,289	1,706	19.77	40.64	26	37	49	0.57	1.16	349,174
Ukraine	4,159	4,505	4,870	17.29	35.53	129	139	151	0.53	1.10	323,202
Thailand	1,909	2,236	2,580	16.31	33.51	69	80	93	0.59	1.20	278,690
Argentina	2,199	2,523	2,865	16.20	33.29	70	81	92	0.52	1.07	312,239
Turkey	1,855	2,178	2,519	16.15	33.19	67	79	91	0.59	1.20	275,553
Sudan	550	850	1,166	14.97	30.77	24	36	50	0.64	1.32	233,104
Spain	1,371	1,650	1,943	13.93	28.62	60	72	85	0.61	1.25	229,705
Kazakhstan	4,179	4,444	4,723	13.24	27.21	161	171	182	0.51	1.05	260,033
Ethiopia	1,268	1,533	1,812	13.22	27.17	47	57	68	0.49	1.02	267,329
France	1,841	2,100	2,373	12.94	26.58	79	90	102	0.56	1.14	232,344
Pakistan	832	1,087	1,357	12.77	26.23	41	53	66	0.62	1.28	205,228
South Africa	817	999	1,191	9.09	18.69	51	63	75	0.57	1.17	159,545
Poland	1,762	1,937	2,121	8.75	17.99	110	121	132	0.55	1.12	160,230
Indonesia	2,441	2,614	2,800	8.67	17.97	128	137	147	0.46	0.94	190,249
Germany	1,824	1,997	2,180	8.66	17.80	112	123	134	0.53	1.09	162,640
Iran	479	645	819	8.27	17.00	34	46	58	0.59	1.21	140,542
Tanzania	726	891	1,065	8.25	16.95	50	62	74	0.57	1.17	144,322
Italy	1,103	1,255	1,414	7.57	15.56	86	98	110	0.59	1.21	128,138
Myanmar	870	988	1,112	5.88	12.09	83	95	106	0.56	1.16	104,439
Romania	937	1,053	1,175	5.79	11.90	90	102	113	0.56	1.15	103,550
Morocco	440	543	651	5.13	10.54	54	67	80	0.63	1.29	81,494
Afghanistan	316	406	502	4.54	9.32	41	52	65	0.58	1.20	77,562
Vietnam	886	975	1,069	4.45	9.15	105	116	127	0.53	1.09	84,234
Algeria	410	499	592	4.41	9.07	57	70	83	0.62	1.27	71,538
Philippines	851	936	1,027	4.29	8.83	104	114	125	0.52	1.08	81,878
Iraq	239	324	414	4.26	8.75	34	47	60	0.61	1.26	69,555
Niger	127	202	280	3.71	7.63	20	32	44	0.58	1.20	63,616
Coate d'Ivoire	330	403	479	3.62	7.44	52	63	75	0.57	1.17	63,560
Ghana	289	358	432	3.48	7.15	47	58	70	0.57	1.16	61,352
Cameroon	327	396	469	3.45	7.10	52	63	74	0.55	1.12	63,147
Mali	105	173	245	3.39	6.97	19	31	44	0.61	1.25	55,655
Uganda	590	655	723	3.24	6.66	95	106	117	0.52	1.08	61,810
Bulgaria	484	549	617	3.22	6.62	88	100	112	0.59	1.20	55,037
Burkina Faso	82	144	209	3.08	6.34	16	29	42	0.62	1.27	49,942
United Kingdom	732	793	858	3.07	6.30	119	129	140	0.50	1.03	61,457
Hungary	410	471	535	3.03	6.24	75	86	98	0.56	1.14	54,528
Syria	203	263	326	2.99	6.14	42	54	67	0.61	1.26	48,738
Belarus	927	985	1,045	2.86	5.87	171	181	193	0.53	1.08	54,268
Bangladesh	693	748	807	2.78	5.71	132	142	154	0.53	1.09	52,551
Cambodia	408	462	520	2.74	5.62	87	99	111	0.59	1.20	46,691
Tunisia	177	232	289	2.72	5.59	41	54	67	0.63	1.30	42,966
Kenya	414	468	525	2.70	5.55	79	89	100	0.51	1.05	52,702

Table S5: Top fifty countries with the highest total annual increment of SOC increase per hectare.

	Total Soil Organic Carbon			Annual Increment		Average per Hectare			Annual Increment		Agricultural Area
	T0	Medium T20	High T20	Medium	High	T0	Medium T20	High T20	Medium	High	
Country	Megatons (t C * 106)					tC/ha					(km2)
Sudan	550	850	1,166	14.97	30.77	24	36	50	0.64	1.32	233,104
Cyprus	12	15	18	0.15	0.32	50	63	76	0.64	1.31	2,421
Botswana	7	10	14	0.18	0.37	24	37	50	0.63	1.30	2,829
Tunisia	177	232	289	2.72	5.59	41	54	67	0.63	1.30	42,966
Gambia	14	18	22	0.20	0.42	42	55	68	0.63	1.30	3,229
Israel	21	26	31	0.25	0.51	53	66	79	0.63	1.30	3,952
India	6,433	8,509	10,699	103.80	213.30	39	52	65	0.63	1.29	1,647,998
Morocco	440	543	651	5.13	10.54	54	67	80	0.63	1.29	81,494
Libya	66	92	120	1.30	2.68	32	44	57	0.62	1.28	20,867
Egypt	408	457	508	2.42	4.98	105	118	131	0.62	1.28	38,877
Pakistan	832	1,087	1,357	12.77	26.23	41	53	66	0.62	1.28	205,228
United States	18,948	21,442	24,071	124.66	256.14	94	107	120	0.62	1.27	2,009,930
Namibia	12	19	26	0.34	0.70	22	34	47	0.62	1.27	5,488
Senegal	137	186	238	2.45	5.03	35	47	60	0.62	1.27	39,540
Burkina Faso	82	144	209	3.08	6.34	16	29	42	0.62	1.27	49,942
Algeria	410	499	592	4.41	9.07	57	70	83	0.62	1.27	71,538
Lebanon	19	22	26	0.18	0.36	66	78	91	0.62	1.27	2,838
Mauritania	6	9	13	0.18	0.36	20	32	45	0.62	1.27	2,857
Jordan	12	17	21	0.22	0.45	34	46	59	0.61	1.26	3,592
Syria	203	263	326	2.99	6.14	42	54	67	0.61	1.26	48,738
Iraq	239	324	414	4.26	8.75	34	47	60	0.61	1.26	69,555
Saudi Arabia	42	64	87	1.10	2.27	23	35	48	0.61	1.26	18,053
Yemen	25	36	49	0.58	1.19	26	39	51	0.61	1.26	9,461
Mali	105	173	245	3.39	6.97	19	31	44	0.61	1.25	55,655
Chad	180	231	284	2.54	5.23	43	55	68	0.61	1.25	41,782
Spain	1,371	1,650	1,943	13.93	28.62	60	72	85	0.61	1.25	229,705
Portugal	321	366	413	2.25	4.63	86	98	111	0.61	1.25	37,153
Albania	62	69	77	0.37	0.77	99	111	124	0.60	1.23	6,206
Turkmenistan	90	115	141	1.23	2.53	44	56	69	0.60	1.23	20,479
Greece	215	246	280	1.59	3.26	80	92	105	0.59	1.22	26,793
Italy	1,103	1,255	1,414	7.57	15.56	86	98	110	0.59	1.21	128,138
Eritrea	14	24	34	0.47	0.96	18	30	42	0.59	1.21	7,954
Iran	479	645	819	8.27	17.00	34	46	58	0.59	1.21	140,542
Uzbekistan	250	299	352	2.47	5.09	59	71	84	0.59	1.21	42,089
Zambia	143	177	213	1.71	3.51	49	61	73	0.59	1.21	29,111
Zimbabwe	143	177	213	1.71	3.51	49	61	73	0.59	1.21	29,111
Somalia	118	148	180	1.50	3.09	46	58	70	0.59	1.21	25,621
Turkey	1,855	2,178	2,519	16.15	33.19	67	79	91	0.59	1.20	275,553
Cambodia	408	462	520	2.74	5.62	87	99	111	0.59	1.20	46,691
Bulgaria	484	549	617	3.22	6.62	88	100	112	0.59	1.20	55,037
Thailand	1,909	2,236	2,580	16.31	33.51	69	80	93	0.59	1.20	278,690
Taiwan	10	11	12	0.06	0.13	87	99	111	0.59	1.20	1,103
Afghanistan	316	406	502	4.54	9.32	41	52	65	0.58	1.20	77,562
Macedonia	47	54	62	0.38	0.78	72	83	96	0.58	1.20	6,501
Niger	127	202	280	3.71	7.63	20	32	44	0.58	1.20	63,616
Angola	147	184	222	1.81	3.72	47	58	71	0.58	1.19	31,399
Croatia	176	197	221	1.10	2.25	92	104	116	0.58	1.18	19,038
Puerto Rico	7	8	9	0.04	0.08	105	117	129	0.57	1.18	712
Tanzania	726	891	1,065	8.25	16.95	50	62	74	0.57	1.17	144,322
Australia	3,418	4,143	4,907	36.23	74.46	54	65	77	0.57	1.17	634,882

Table S6: Top forty countries with the highest total area of high SOC soils.

High SOC Soils	Total Soil Organic Carbon	Average per Hectare	Agricultural Area
	T_0	T_0	
Country	Megatons (t C * 106)	tC/ha	km^2
Indonesia	3,385	256	132,378
Russia	2,013	279	72,203
Malaysia	562	292	19,251
New Zealand	246	194	12,710
Canada	254	233	10,887
Norway	187	230	8,135
Brazil	82	105	7,783
Colombia	111	177	6,272
Chile	112	189	5,908
Ecuador	104	178	5,823
Finland	125	265	4,711
Japan	105	223	4,697
United States	221	526	4,196
Peru	64	180	3,569
Sweden	86	251	3,420
Ethiopia	32	128	2,468
Democratic Republic of the Congo	40	173	2,316
Australia	38	169	2,242
Kazakhstan	80	395	2,014
Vietnam	56	283	1,997
Papua New Guinea	36	187	1,923
Rwanda	24	155	1,536
China	42	374	1,133
Philippines	26	243	1,081
Belarus	33	393	843
Ireland	21	261	811
Cameroon	11	134	806
India	19	234	805
Tanzania	8	104	768
Thailand	20	393	516
Myanmar	15	315	478
Bangladesh	18	392	465
Ukraine	16	357	459
North Korea	10	238	419
Argentina	8	212	395
Kenya	6	145	393
Bhutan	6	183	354
Guatemala	6	181	337
United Kingdom	8	255	303
Cambodia	13	417	301

Supplementary Figures:

Figure S1: High soil organic carbon (SOC) and sandy soils. Distribution of high SOC and sandy soils which were excluded from the analysis, i.e. soils with a bulk density of less the 1.0 g/cm^3, and/or SOC greater than 400 t C/ha in the top 15cm, and soils with a sand content equal to or greater than 85 %. Maps were produced based upon a geospatial analysis of datasets from the SoilsGrids250 database (ISRIC; Hengl et al, 2017), using ESRI ArcGIS software (version 10.3; www.esri.com).

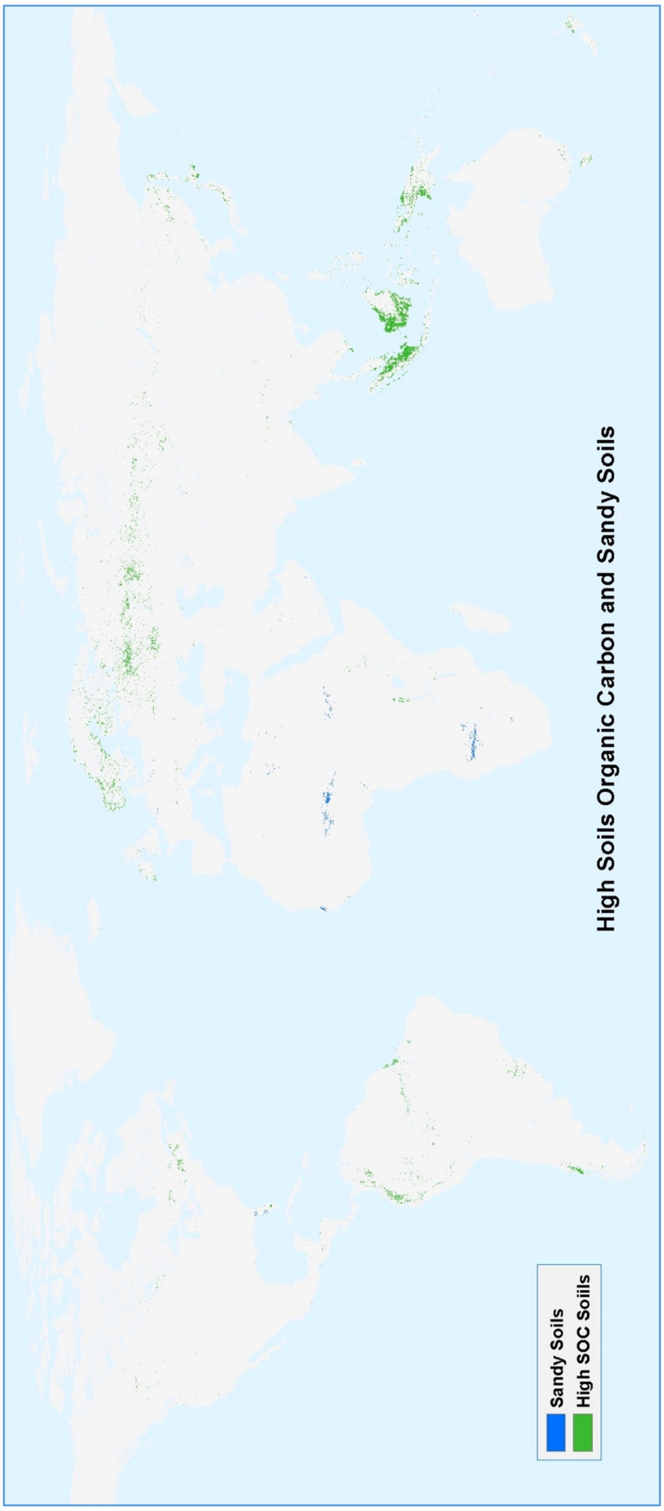

Figure S2: Annual increase in soil organic carbon (SOC) – Africa. Annual increase in soil organic carbon (SOC) in the top 30 cm, on all available cropland soils globally (i.e. those not excluded from the analysis as high SOC or sandy soils) under the medium scenario (i.e. an increase in percent SOC of 0.27 over 20 years). Maps were produced based upon a geospatial analysis of datasets from the SoilsGrids250 database (ISRIC; Hengl et al, 2017), using ESRI ArcGIS software (version 10.3; www.esri.com).

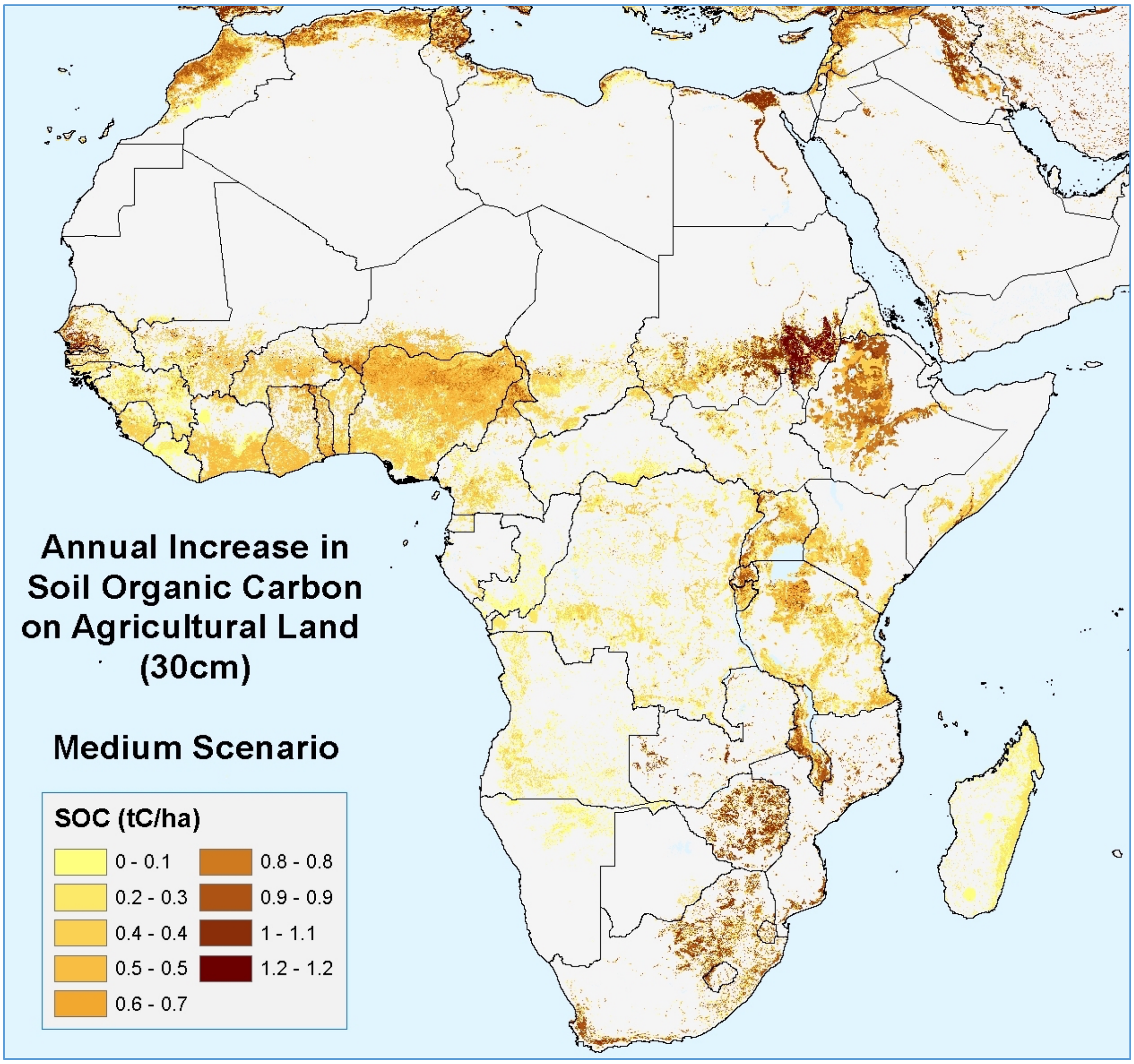

Figure S3: Annual increase in soil organic carbon (SOC) – South America. Annual increase in soil organic carbon (SOC) in the top 30 cm, on all available cropland soils globally (i.e. those not excluded from the analysis as high SOC or sandy soils) under the medium scenario (i.e. an increase in percent SOC of 0.27 over 20 years). Maps were produced based upon a geospatial analysis of datasets from the SoilsGrids250 database (ISRIC; Hengl et al, 2017), using ESRI ArcGIS software (version 10.3; www.esri.com).

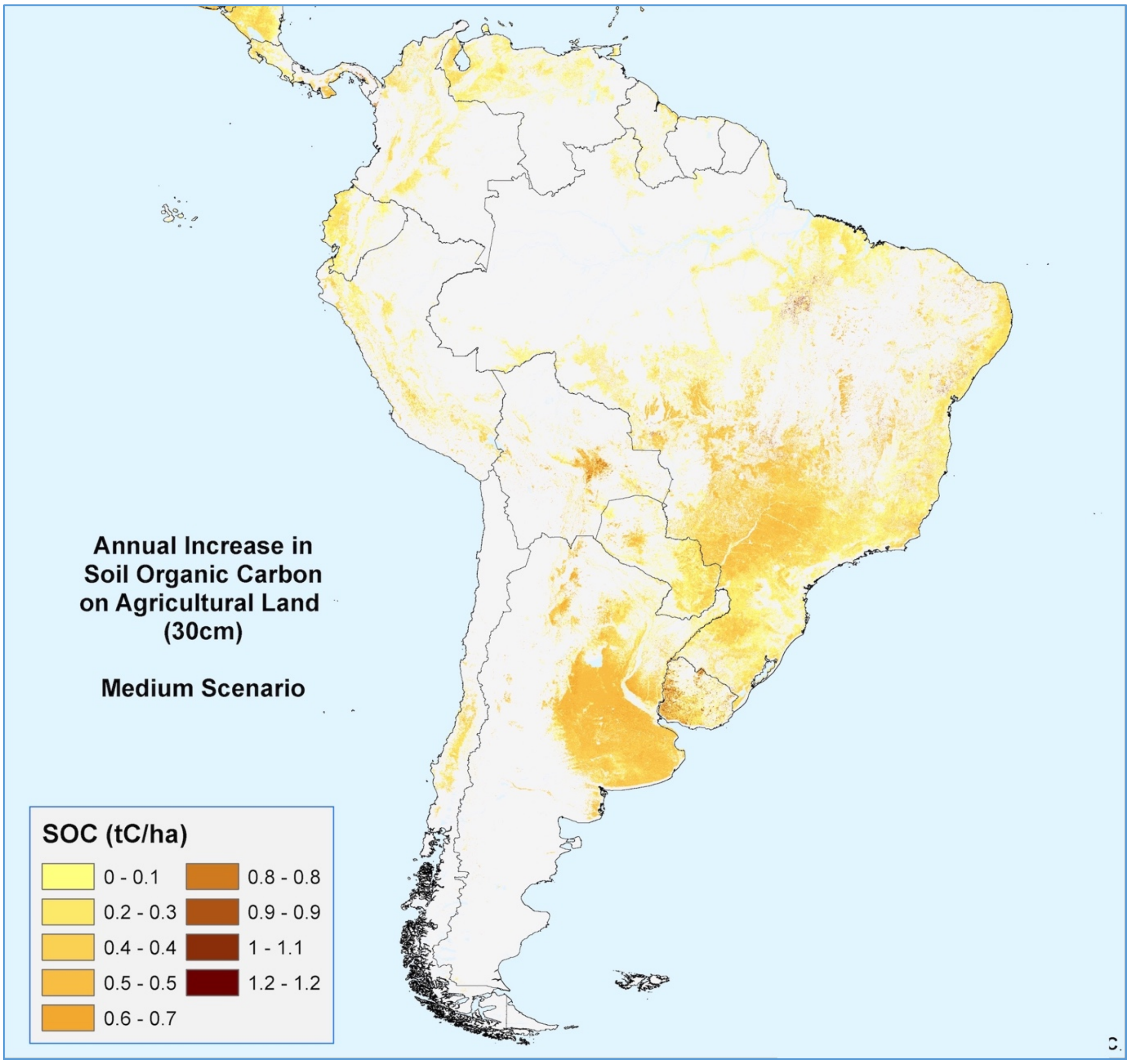

Figure S4: Annual increase in soil organic carbon (SOC) – South Asia. Annual increase in soil organic carbon (SOC) in the top 30 cm, on all available cropland soils globally (i.e. those not excluded from the analysis as high SOC or sandy soils) under the medium scenario (i.e. an increase in percent SOC of 0.27 over 20 years). Maps were produced based upon a geospatial analysis of datasets from the SoilsGrids250 database (ISRIC; Hengl et al, 2017), using ESRI ArcGIS software (version 10.3; www.esri.com).

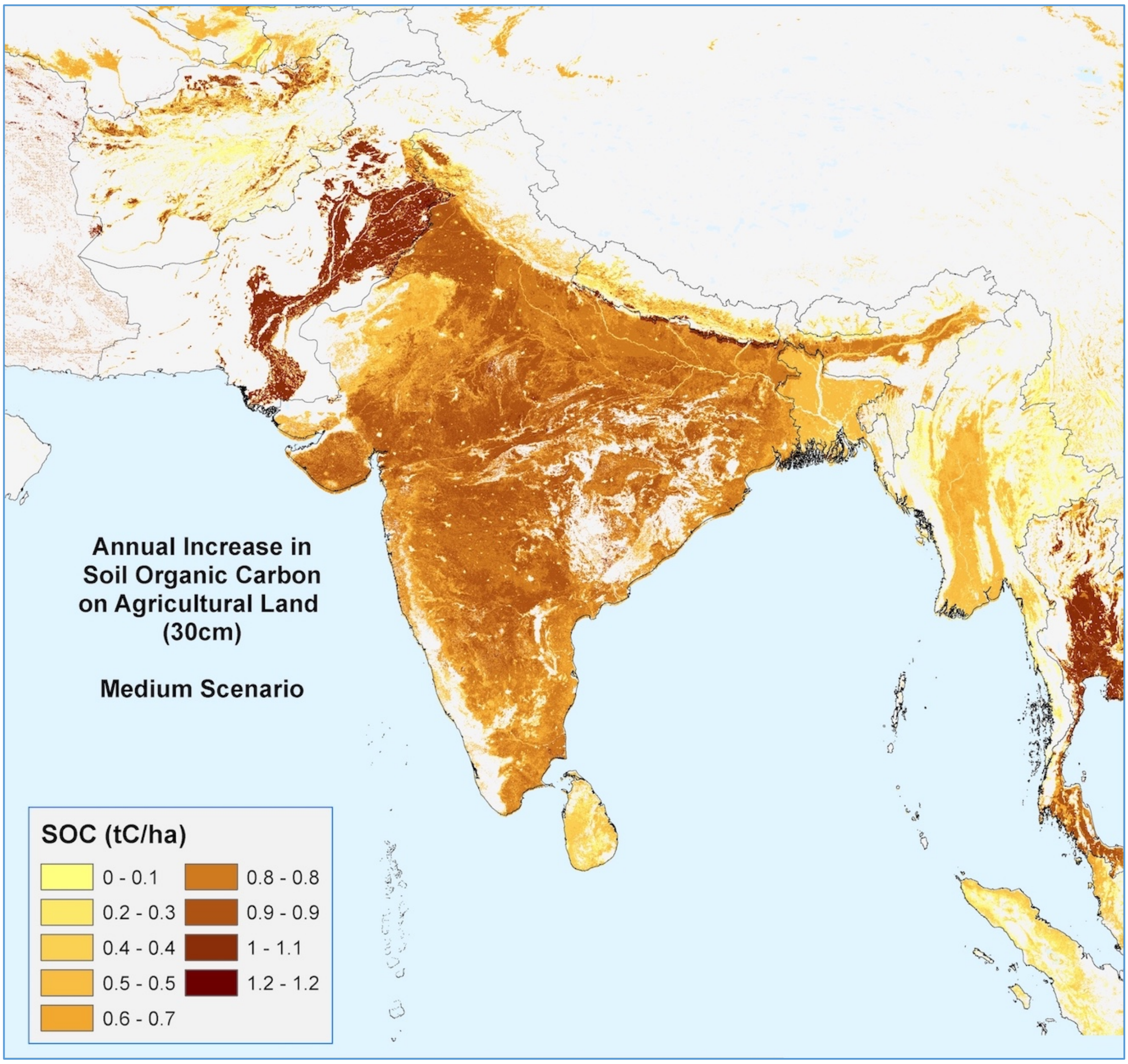

Figure S5: Annual increase in soil organic carbon (SOC) – Southeast Asia. Annual increase in soil organic carbon (SOC) in the top 30 cm, on all available cropland soils globally (i.e. those not excluded from the analysis as high SOC or sandy soils) under the medium scenario (i.e. an increase in percent SOC of 0.27 over 20 years), and distribution of the high SOC soils. Maps were produced based upon a geospatial analysis of datasets from the SoilsGrids250 database (ISRIC; Hengl et al, 2017), using ESRI ArcGIS software (version 10.3; www.esri.com).

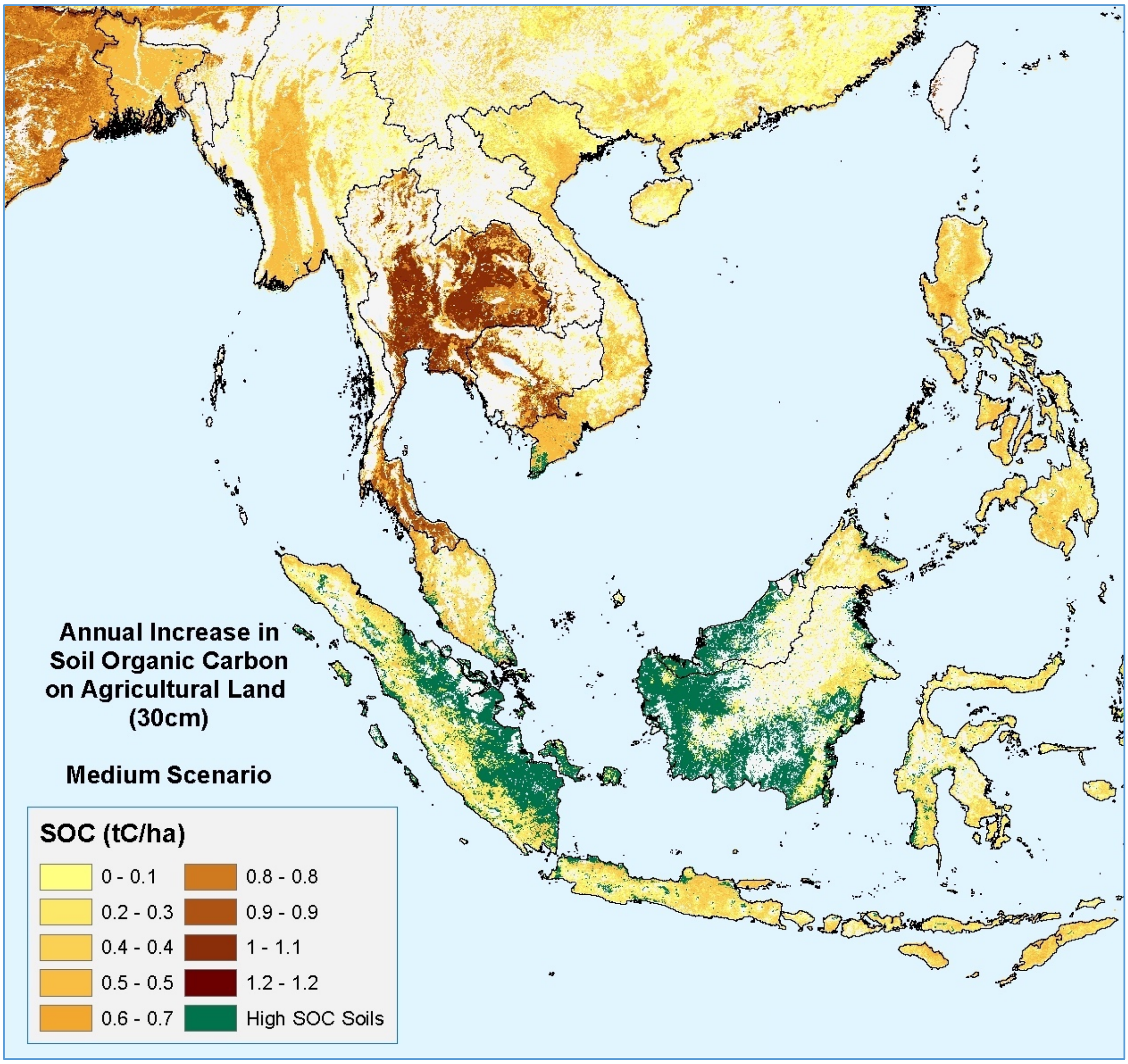

Figure S6: Annual increase in soil organic carbon (SOC) – East Asia. Annual increase in soil organic carbon (SOC) in the top 30 cm, on all available cropland soils globally (i.e. those not excluded from the analysis as high SOC or sandy soils) under the medium scenario (i.e. an increase in percent SOC of 0.27 over 20 years). Maps were produced based upon a geospatial analysis of datasets from the SoilsGrids250 database (ISRIC; Hengl et al, 2017), using ESRI ArcGIS software (version 10.3; www.esri.com).

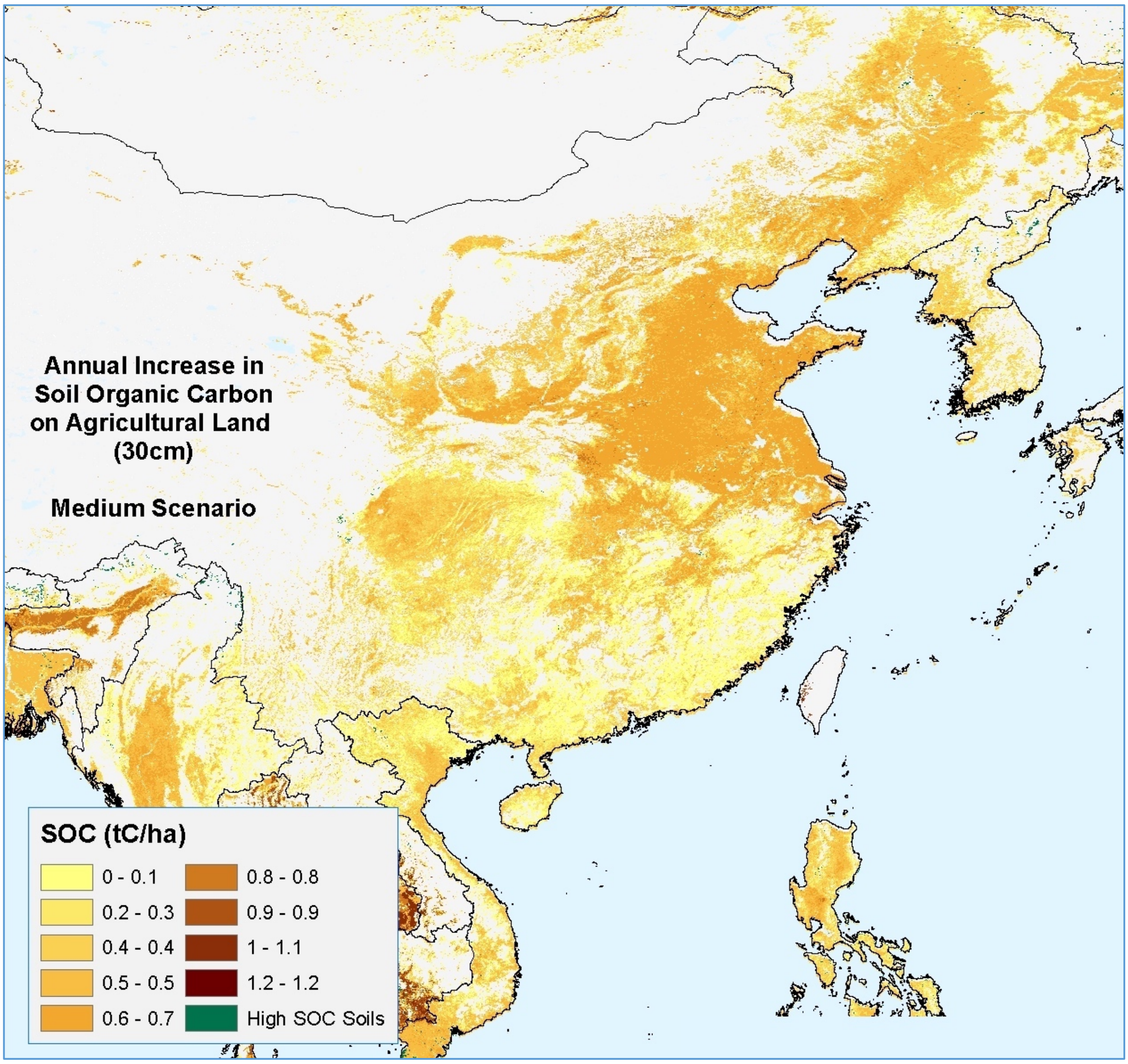

Figure S7: Annual increase in soil organic carbon (SOC) – Europe. Annual increase in soil organic carbon (SOC) in the top 30 cm, on all available cropland soils globally (i.e. those not excluded from the analysis as high or sandy SOC soils) under the medium scenario (i.e. an increase in percent SOC of 0.27 over 20 years). Maps were produced based upon a geospatial analysis of datasets from the SoilsGrids250 database (ISRIC; Hengl et al, 2017), using ESRI ArcGIS software (version 10.3; www.esri.com).

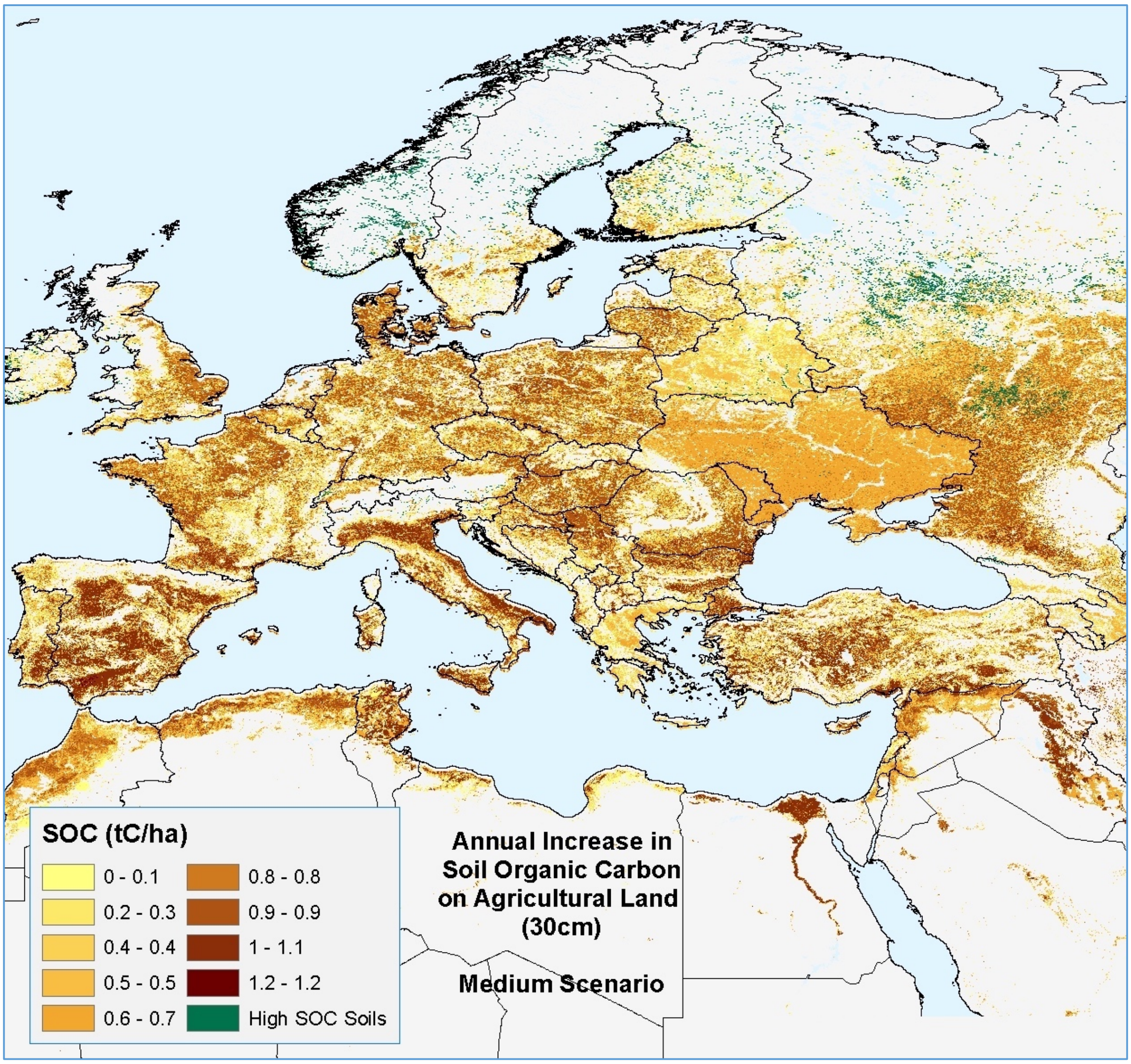

Figure S8: Annual increase in soil organic carbon (SOC) – North America. Annual increase in soil organic carbon (SOC) in the top 30 cm, on all available cropland soils globally (i.e. those not excluded from the analysis as high SOC or sandy soils) under the medium scenario (i.e. an increase in percent SOC of 0.27 over 20 years). Maps were produced based upon a geospatial analysis of datasets from the SoilsGrids250 database (ISRIC; Hengl et al, 2017), using ESRI ArcGIS software (version 10.3; www.esri.com).

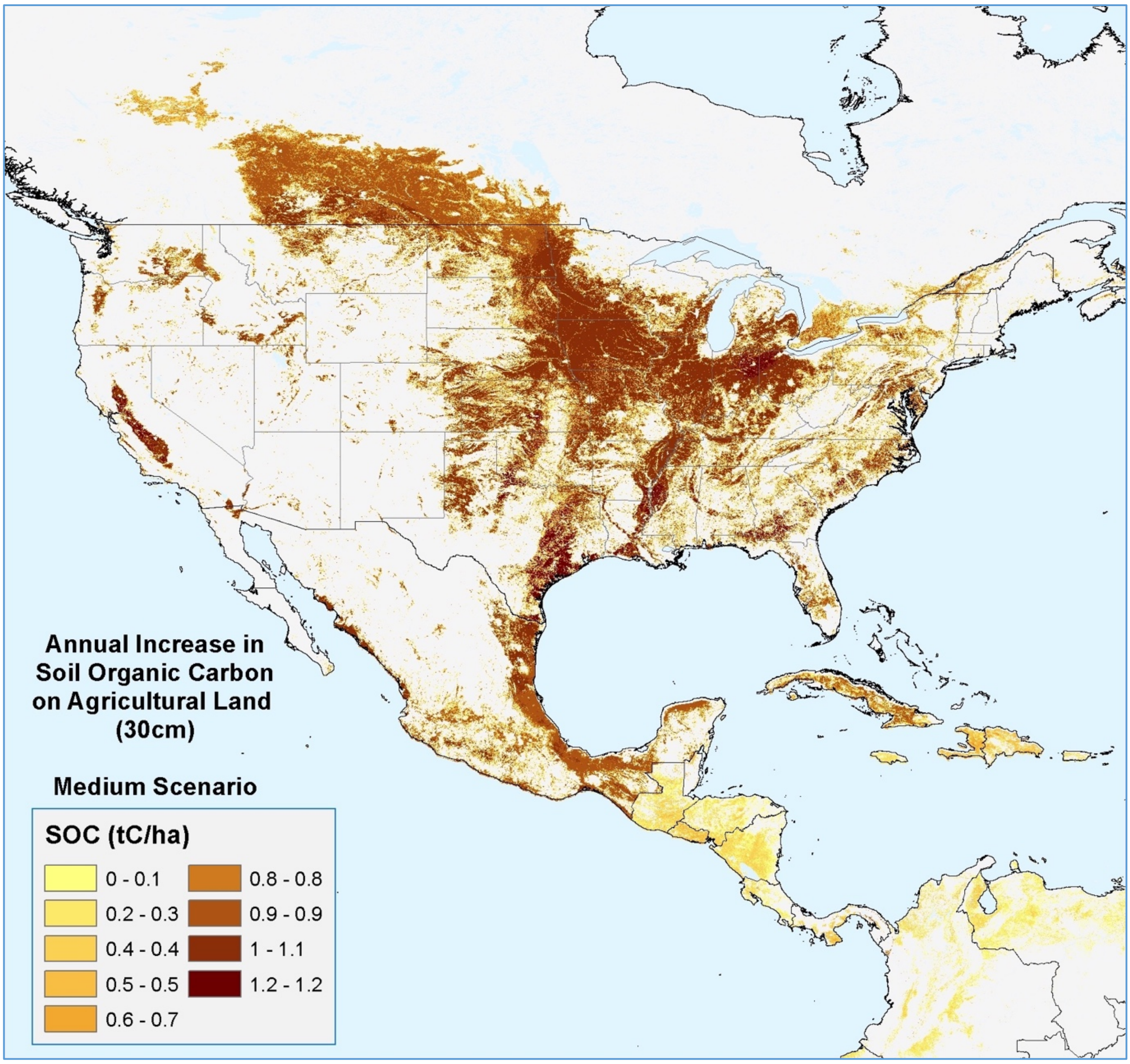

Figure S9: Annual increase in soil organic carbon (SOC) – Australia. Annual increase in soil organic carbon (SOC) in the top 30 cm, on all available cropland soils globally (i.e. those not excluded from the analysis as high SOC or sandy soils) under the medium scenario (i.e. an increase in percent SOC of 0.27 over 20 years). Maps were produced based upon a geospatial analysis of datasets from the SoilsGrids250 database (ISRIC; Hengl et al, 2017), using ESRI ArcGIS software (version 10.3; www.esri.com).

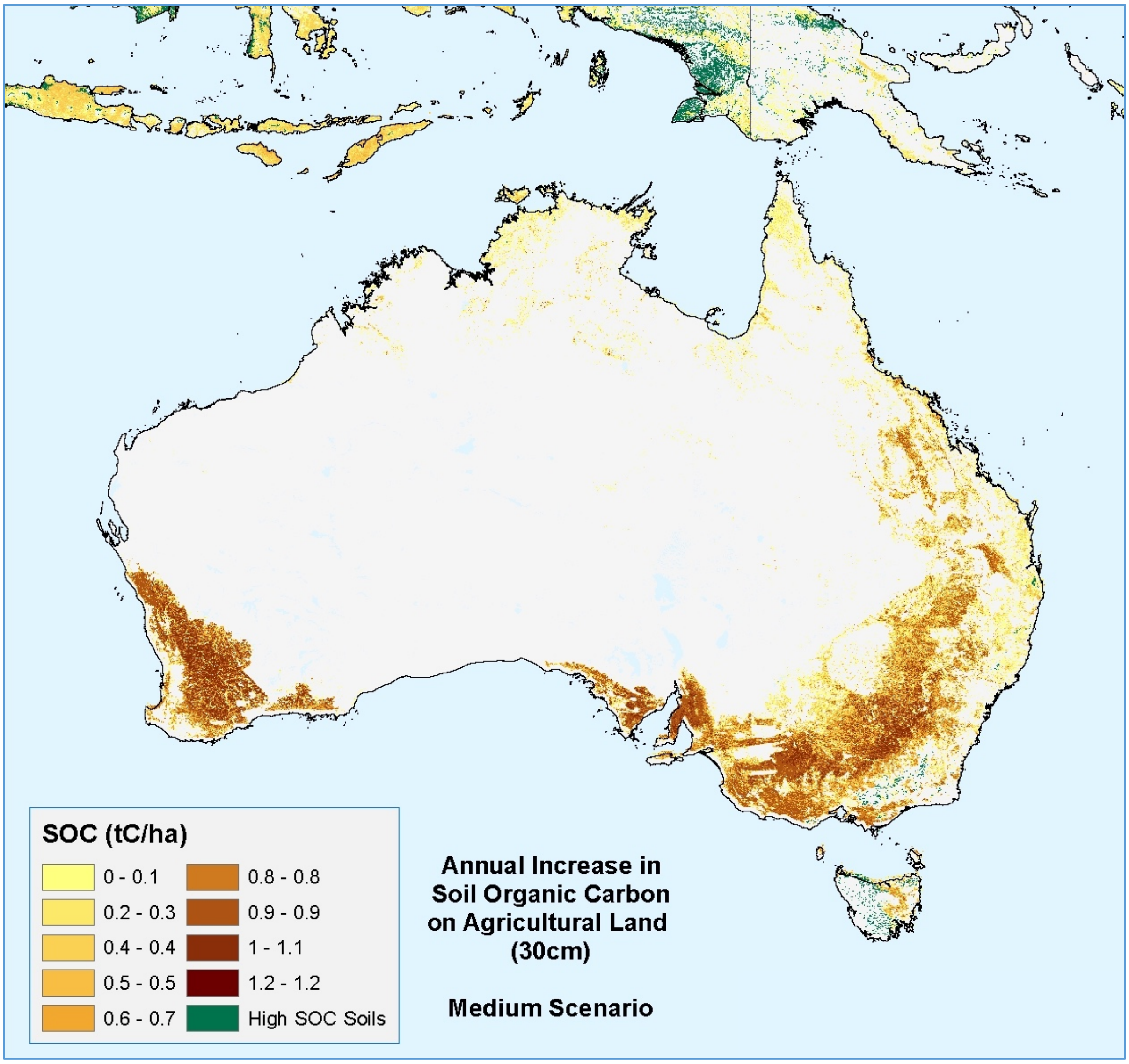

References

1. Sommer, R. & Bossio, D. Dynamics and climate change mitigation potential of soil organic carbon sequestration. *J. Environ. Manage.* **144,** 83–87 (2014).
2. Hengl, T. *et al.* SoilGrids250m: Global gridded soil information based on machine learning. *PLoS ONE* **12,** e0169748 (2017).
3. Latham, J., Cumani, R., Rosati, I. & Bloise, M. *FAO Global Land Cover (GLC-SHARE) Beta-Release 1.0 Database, Version 1.0 - 2014*. 1–40 (Land and Water Division, FAO, 2014).
4. GADM. GADM Database of Global Administrative Areas. (2005).
